# The Cardiac Stress Response Factor Ms1 Can Bind to DNA and Has a Function in the Nucleus

**DOI:** 10.1371/journal.pone.0144614

**Published:** 2015-12-14

**Authors:** Mariola Zaleska, Claudia Fogl, Ay Lin Kho, Abdessamad Ababou, Elisabeth Ehler, Mark Pfuhl

**Affiliations:** 1 Cardiovascular and Randall Division, King's College London, London, United Kingdom; 2 Department of Biochemistry, University of Leicester, Lancaster Road, Leicester, United Kingdom; 3 Department of Pathology, University of Cambridge, Tennis Court Road, Cambridge, United Kingdom; Florida International University Bimolecular Sciences Institute, UNITED STATES

## Abstract

Ms1 (also known as STARS and ABRA) has been shown to act as an early stress response gene in processes as different as hypertrophy in skeletal and cardiac muscle and growth of collateral blood vessels. It is important for cardiac development in zebrafish and is upregulated in mouse models for cardiac hypertrophy as well as in human failing hearts. Ms1 possesses actin binding sites at its C-terminus and is usually found in the cell bound to actin filaments in the cytosol or in sarcomeres. We determined the NMR structure of the only folded domain of Ms1 comprising the second actin binding site called actin binding domain 2 (ABD2, residues 294–375), and found that it is similar to the winged helix-turn-helix fold adopted mainly by DNA binding domains of transcriptional factors. *In vitro* experiments show specific binding of this domain, in combination with a newly discovered AT-hook motif located N-terminally, to the sequence (A/C/G)AAA(C/A). NMR and fluorescence titration experiments confirm that this motif is indeed bound specifically by the recognition helix. In neonatal rat cardiomyocytes endogenous Ms1 is found in the nucleus in a spotted pattern, reminiscent of PML bodies. In adult rat cardiomyocytes Ms1 is exclusively found in the sarcomere. A nuclear localisation site in the N-terminus of the protein is required for nuclear localisation. This suggests that Ms1 has the potential to act directly in the nucleus through specific interaction with DNA in development and potentially as a response to stress in adult tissues.

## Introduction

Ms1/STARS is a stress response protein of the cardiac and skeletal muscle (recently reviewed in [1]). It was discovered as a protein overexpressed in cardiomyocytes early in the hypertension model of rat aortic banding [2] and as a novel gene expressed exclusively in the mouse heart tube [3]. It was shown to be important for the maintenance of cardiac development in zebrafish [4] and is upregulated in mouse models of cardiac hypertrophy and in failing human hearts [5]. Its expression appears to be regulated by MEF-2, a myogenic transcription factor also involved in stress response signalling [5] as well as GATA4 [6]. Overexpression of Ms1 in cardiomyocytes sensitises the heart to pressure overload and calcineurin signalling, leading to an exaggerated deterioration of cardiac function. In H9c2 cells, overexpression of Ms1 induces cellular hypertrophy and protects against apoptosis through alteration of the expression pattern of genes regulated by the MRTF/SRF pathway [7]. Ms1 is a highly conserved protein present in all eukaryotes and in vertebrates is expressed mainly in cardiac and skeletal muscle. Recently, it was also found in endothelial tissue involved in the formation of collateral blood vessels [8].

The size of the protein increased in the course of evolution from a short, 81 residue version called COSTARS [9] in the smallest known eukaryotes via a version of about 150 residues in insects and nematodes and just under 400 amino acids in vertebrates [10] (S1 Fig). The protein was shown to bind to F-actin [3,10], which was increased by its interaction with ABLIM-1/2 [11]. We were able to show that only its highly conserved C-terminus assumes a well-folded structure, while the rest of the protein is highly flexible and unfolded [10]. The folded C-terminal domain, termed ABD2 (actin binding domain 2, residues 294–375), contains one of two independent F-actin binding sites, immediately preceded by a second one, termed ABD1 (residues 193–295), of higher affinity [10]. Ms1 is assumed to cooperate with Rho and other upstream factors in stabilising cellular F-actin. Stabilising F-actin leads to a reduction in the free G-actin pool and consequently the release of the myocardin-regulated SRF cofactors MRTF-A and -B for translocation to the nucleus where they can influence transcriptional regulation [3,5]. In summary, Ms1/STARS has somewhat contrasting functions: on the one hand, it has an essential function in cardiogenesis and is even cardioprotective. On the other hand, its overexpression renders cardiomyocytes susceptible to pressure overload and heart failure. The current view is that Ms1 acts as an indirect regulator of transcription by controlling the nuclear translocation of MRTFs via F-actin polymerisation.

Here, we determined the structure of its only folded domain, ABD2, to better understand its actin binding mechanism. As in the case of the homologous protein COSTARS [12], we found that it has a winged helix-turn-helix fold, most commonly found in the DNA binding domain of transcription factors. We were able to show that an extended construct of ABD2, including an AT-hook DNA binding motif, is indeed able to bind to DNA in a sequence specific manner. Cell biology studies show that the protein is able to reach the nucleus based on NLS dependent transport in a tightly regulated fashion. This suggests that in addition to the established function via a rho/actin/MRTF/SRF pathway Ms1 can also act directly in the nucleus.

## Materials and Methods

All animals used in this study received humane care under the guidelines of the Animals (Scientific Procedures) Act, 1986

### Sequence analysis

The alignment for full length Ms1 was based on 3 cycles of Ψ-Blast alignments using the default parameters of the EBI web server (www.ebi.ac.uk) and a cutoff value of 2 10^−20^. After each cycle sequence hits were inspected manually and only complete proteins were carried over to the next cycle while proteins characterised as fragments were removed to avoid false identification of shorter Ms1 versions. Also sequences, which were significantly longer (>450aa) or had a distinctly different description were removed. A representative subset of sequences was extracted, aligned using the default settings of clustalw and coloured with clustalx as described previously [13]

Experimentally validated phosphorylation sites were searched on the RegPhos [14] and PhosphoSitePlus [15] web servers. Nuclear localisation sequences (NLS) were searched with PredictNLS [16].

### Protein preparation

ABD2 (residues 294–375) and extended ABD2 (residues 270; 281 and 287–375) as well as N-terminus (residues 1–99) and ABD1 (residues 193–295) from rat Ms1 were cloned into pLEICS-07 vector, which contains an N-terminal, TEV cleavable, poly-histidine tag (kindly provided by Dr. Xiaowen Yang, PROTEX, University of Leicester). They were expressed in *E*.*coli* strain BL21* (Invitrogen) and purified as described previously [10]. The protein was dialysed into appropriate buffer (NMR, EMSA or SELEX buffer, see below) and concentrated using a viva-spin 20 concentrator with a MES membrane and a 3kD molecular weight cutoff.

### NMR spectroscopy & structure determination

All experiments were performed at 298 K and pH 7.2 with a range of concentrations between 188 μM and 500 μM in a buffer consisting of 20mM sodium phosphate pH 7.0, 50mM NaCl, 2mM DTT, 0.02% NaN3, on Bruker Avance spectrometers at 500, 600, 700 and 800 MHz all equipped with cryoprobes. Further details of the individual experiments used can be found in PDB entry 2KRH.

CCPN Analysis [17] was used to analyse the spectra, pick the peaks, perform the sequence specific assignment and to extract distance constraints for structure calculation.

Dihedral angle constraints were extracted from chemical shifts with TALOS [18]. They were combined with NOE distance restraints and used in CYANA 2.1 [19] to calculate the structure of ABD2 using default settings and the NOA protocol to assign ambiguous NOEs. The NMR constraints and statistics of the structure calculation are summarised in Table 1. Further details of the individual experiments used to collect constraints used in the structure calculation can be found in PDB entry 2KRH. Structure similarities were analysed with the DALI server [20,21].

**Table 1:** Structure calculation statistics of ABD2.

**Input Constraints**	
Dihedral Angle Constraints	130
NOE-derived Distances	1679
Hydrogen Bond Constraints	32
**Structure Statistics**	
Backbone RMSD	0.54 Å
Heavy atom RMSD	1.30 Å
Residues in core region of the Ramachandran plot	83.3%
Residues in allowed region of the Ramachandran plot	16.4%
Average / maximal violation of NOE constraints	0.0222 / 0.135
Average / maximal violation of dihedral constraints	0.3669 / 0.4822

### DNA binding assays & SELEX

Protein in EMSA buffer (20 mM PO_4_, 100 mM NaCl, 0.01% Triton X-100, pH 7.5) was incubated with 1 μM or 2 μM of DNA in a total volume of 20 μl. Protein concentration ranged from 1 μM to 100 μM depending on the experiment. FOXO3a, an extensively characterised transcription factor was used as a positive control [22]. As a DNA probe, a random dsDNA library (Gene Link) or ds oligonucleotides (Biomers) were used. The dsDNA library was prepared as previously described [23], whilst ds oligonucleotides were prepared by mixing equal molar amounts of single stranded complementary oligonucleotide and heating for 10 minutes at 95°C, followed by a gradual cooling to room temperature in a thermocycler for 5 hours. The EMSA samples were incubated for 30 minutes at room temperature and then 2 μl of 80% glycerol was added and the samples were loaded onto a 1.6% agarose gel prepared with SYBRSafe stain (Invitrogen). The gel was run for 40 minutes at 80V and the samples were visualised under UV light.

To identify sequences bound by ABD2 or the extended construct, a SELEX experiment was performed as previously described [23], except that a shorter library was used (containing N_18_ in the random region) and that the final pool was cloned into pCR 2.1 vector using the TA cloning kit (Invitrogen). To identify the positive clones, colony PCR was performed and the positive PCR products were sequenced (Beckman Coulter Genomics). The sequencing data was further analysed using the MEME suite [24] (http://meme.nbcr.net)

### Antibody production

To generate antibodies against Ms1, 3 different Ms1 constructs were prepared: N-terminus (1–99 aa), ABD1 (193–295 aa) and ABD2 (294–375 aa). They were expressed and purified as described earlier [10]. 10 mg/ml of each protein was sent for rabbit immunisation to 2 different companies (Absea, China and Bioscience, Germany). The obtained sera were tested using Western blot and immunofluorescence. The serum that gave the best results (aABD2chn) was further validated using overexpression studies and immunofluorescence. As a control for specificity the antibody was pre absorbed with the immunising antigen and then used on western blots and in immunofluorescence. No signal was detected in both with pre absorbed antibody (aABD2chn antibody validation–S4 and S5 Figs).

### Transfection constructs and point mutation

Myc-tagged mouse Ms1 cloned into the mammalian expression vector pcDNA3.1(+) was kindly provided by Dr. A. Koekemoer, University of Leicester. Point mutations were created using the QuikChange Site Directed Mutagenesis kit (Agilent Technologies). Primers carrying the desired mutation were designed using the QuikChange Primer Design software (www.genomics.agilent.com/primerDesignProgram.jsp). Mutagenesis was performed following the manufacturer’s protocol. DNA from successful colonies was sent for sequencing (Source Bioscience, UK) and positive clones were stored at -20°C.

### Western blot

Protein samples from NRC and ARC cultures were prepared by scraping cells from the dish using a cell scraper and by subsequent lysis in a protein loading buffer at 90°C for 10 minutes. Protein samples were separated by SDS-PAGE using pre-cast NuPage 4–12% Bis Tris gels (Invitrogen) and blotted onto PVDF membrane (Milipore). After the transfer, membranes were blocked in TBST buffer supplemented with 5% milk (Sigma-Aldrich) for 1 hour at room temperature, followed by overnight incubation with primary antibody at 4°C. The following antibodies diluted in TBST + 5% milk were used: polyclonal rabbit anti-Ms1 (aABD2chn, 1:25000) polyclonal rabbit anti-actin (Sigma-Aldrich, 1:200), polyclonal rabbit anti-lamin A (Sigma-Aldrich, 1:200). After that, membranes were washed 3 times in TBST + 5% milk and HRP-conjugated anti-rabbit or anti-mouse Igs secondary antibody was added (1:5000 dilution, Santa Cruz). The secondary antibody was incubated for 1 hour at room temperature, followed by 3 washes in TBST buffer. To detect the antibody, chemiluminescent reagent (Invitrogen) was added and the blots were exposed to X-ray film (Fuji Film, RX NIF).

### Cells

For the antibody validation, Hela cells were used (kindly provided by Dr. Stephen Terry). To study Ms1 cellular localisation as well as for antibody validation, NRC (Neonatal Rat Cardiomyocyte) and ARC (Adult Rat Cardiomyocyte) primary cell cultures were used. NRCs and ARCs were produced from Sprague-Dawley rats as described previously [25,26]. Rats were aged 1–2 days for NRCs and 6 months for ARCs.

### Cell Transfection

Hela cells as described in [27] were plated on 12 mm glass cover slips in 6-well plate format. They were transfected with myc-tagged Ms1 construct using Lipofectamine 2000 (Invitrogen). For each well with a cover slip a mixture of 1μg of plasmid plus 5 μl of Lipofectamine in DMEM medium was added. Cells were incubated overnight, and the next day the medium was changed into fresh DMEM medium with 10% FCS. The transient transfection was carried out for the next 48 hours. After that, cells were fixed with 4% paraformaldehyde (Agar Scientific) in PBS for 10 min. for immunofluorescence or lysed for Western blot analysis.

NRCs as described in [28] were plated onto collagen-coated 35 mm culture dishes (PureCol; Inamed Biomaterials). Transfection was done as for the Hela cells, except that Escort III (Sigma-Aldrich) and different media depending on the experiment were used. To study cells in the presence of a hypertrophic stimulus, the next day, the plating medium (68% DME, 16% medium M199, 10% horse serum, 5% fetal calf serum, 4 mM glutamine) was changed to maintenance medium (78% DME, 20% medium M199, 4% horse serum, 1% penicillin–streptomycin, 4 mM glutamine, and 0.1 mM phenylephrine [Sigma-Aldrich]). To study cells in the absence of a hypertrophic stimulus, plating medium was used for the whole experiment. After 48 hours cells were fixed with 4% PFA in PBS for immunofluorescence or lysed for Western blot analysis.

### Cellular fractionation

Cellular fractionation was performed using the Subcellular Protein Fractionation kit for Cultured Cells (Thermo Scientific) following the manufacturer’s protocol. Cellular fractionation was performed for Hela, NRC and ARC cells yielding cytoplasmic, membrane, soluble nuclear, chromatin-bound and cytoskeletal protein fractions. Later on each fraction was analysed for the presence of Ms1 protein using Western blot.

### Immunofluorescence

The following cell types were used for immunofluorescence: Hela, NRCs and ARCs. The immunofluorescence protocol used was the same for all of them except that Hela cells were plated on glass coverslips and NRCs and ARCs onto collagen-coated 35 mm culture dishes. Briefly, cells were fixed for 10 minutes with 4% paraformaldehyde in PBS and permeabilised for 5 minutes with 0.1% TX-100 in PBS, following by 1 wash in PBS. After that, they were blocked in the blocking reagent (Hela in 1%BSA in PBS, NRCs and ARCs in MAXblock, Activ Motif) for 1 hour at room temperature. Later, the cells were incubated in the primary antibody solution for 1 hour at room temperature (ARCs were incubated overnight at 4°C), followed by 3 washes in PBS and incubation in secondary antibody solution for 1 hour at room at room temperature (or 5 hours at room temperature for ARCs). Primary and secondary antibodies were diluted in 1% BSA in PBS. The following primary antibodies were used: polyclonal rabbit anti-Ms1 (aABD2chn, 1:2000), monoclonal mouse anti-sarcomeric alpha-actinin, clone EA-53 (Sigma-Aldrich, 1:500), monoclonal mouse anti-c-myc (Roche, 1:500). Polyclonal rabbit anti-titin (M8 epitope) was kindly donated by Mathias Gautel (King’s College London, UK). Primary antibodies were visualised using Alexa488-conjugated anti-mouse Igs and Cy-5-conjugated anti-rabbit Igs (Jackson ImmunoResearch Laboratories, Inc.) To visualise actin AlexaFluor488- or AlexaFluor568-conjugated phalloidin was used (Invitrogen). For the nucleus staining DAPI (Sigma-Aldrich) was used. Cells were mounted in 0.1 M Tris-HCl/glycerol (3:7) and 50 mg/ml N-propylgallate, pH 9.5 and photographed using a Nikon microscope at 60x magnification and a nominal aperture of 1.4 (Ar1 confocal). Images were processed using ImageJ software.

## Results

### Structure of ABD2

The C-terminal domain of Ms1, which has a high sequence similarity to the small, homologous protein COSTARS [9], was shown to be the only part of full length Ms1 to be folded on its own [10]. It folds into a compact monomer as shown by size exclusion chromatography [10]. Analysis of R1/R2 ratios [29] from ^15^N relaxation experiments for all rigid amino acids results in a τ_c_ value of 7.4 +- 0.3 ns which is in good agreement with the value expected for a spherical protein of 10 kD and its hydration shell. The structure was determined using standard NMR methods and has been submitted to the PDB, accession code 2KRH.

**Fig 1:**
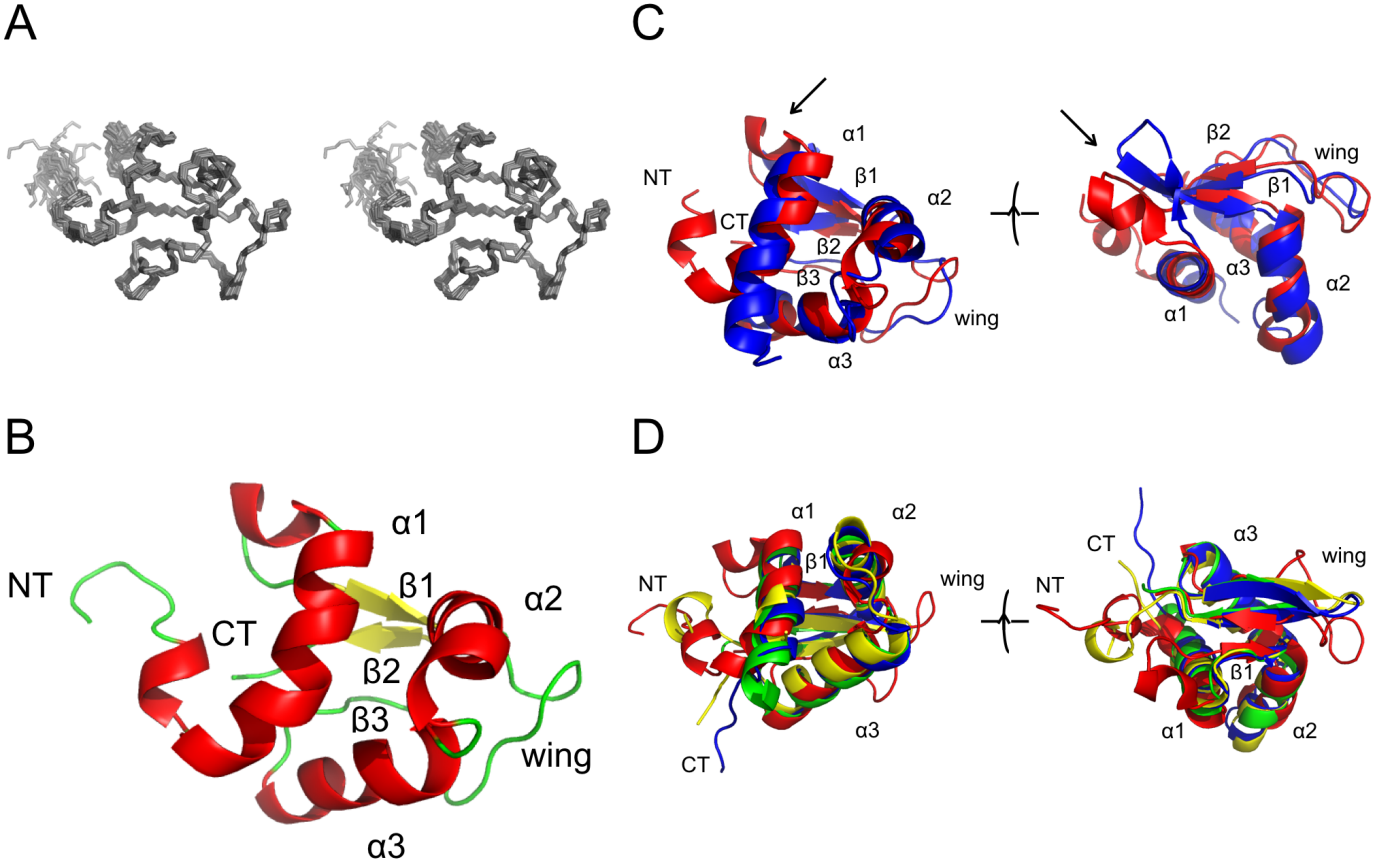
Structure determination of rat ABD2. A: Stereo view of the family of the best 20 structures superimposed on the lowest energy structure. B: Secondary structure of the lowest energy structure in the same orientation as in A. Key structural features are labeled, helices are coloured red and strands are coloured yellow. C: Structural superposition of Ms1 ABD2 (red, PDB ID 2KRH) and COSTARS (blue, PDB ID 2L2O). Shown in the same orientation as in B as well as rotated about 90° around the horizontal axis to reveal the structural differences in the segment connecting α1 and β1 (arrow). D: Structural superposition of Ms1 ABD2 (red) on the three top hits of the DALI search in the PDB (1HR3 (green), 1SAX (blue), 1SD4 (yellow); for a list of the top 50 DALI hits see S3 Fig).

The structure is shown in Fig 1. The family of structures shows good agreement for all secondary structure elements and most loops. Some higher divergence is seen for the N-terminus until the start of the first helix and loop 2. These regions of increased divergence show higher levels of local mobility in ^15^N relaxation experiments (S2 Fig). The overall secondary structure is comprised of three α-helices and a three stranded β-sheet making for a small and compact overall shape.

The structure of Ms1/STARS ABD2 is globally very similar to that of its small homologue COSTARS [9,12]. A DaliLite comparison gives a Cα RMSD of 3.0 Å and a Z-score of 8.3 despite a low sequence identity of only 34%. The only significant difference in the structures is found in the loop between helix 1 and strand 1 where COSTARS has an additional short β-strand whereas ABD2 has a short α-helix (see arrow in Fig 1C).

No proteins outside the Ms1 family with significant sequence similarity could be identified by sequence searches. As a result, structural similarity searches with DALI [20] were performed to gain more insight into the function of ABD2. Despite the initial identification of ABD2 as an actin binding domain [10] no actin binding domain was identified in the top hits produced by Dali (S3 Fig). Instead, the structure has a substantial similarity to winged helix-turn-helix domains, a subset of the helix-turn-helix DNA binding domain family (Fig 1D). The basic helix-turn-helix domain is a three helix core, which binds to DNA with its third helix [30,31]. The major distinguishing feature of a winged helix-turn-helix protein is the presence of a C-terminal β-strand hairpin unit (the wing in the name of the protein domain family) [32,33]. This wing sometimes packs into the shallow cleft in the open core of the domain [34]; however, this packing is absent in ABD2.

In a typical domain containing a winged helix-turn-helix motif, the N-terminus contains three α-helices (H1, H2 and H3) and three β-strands (S1, S2, and S3). In ABD2 these are found at the following residues, H1 = R302 to M314, H2 = F325 to S338, H3 = V341 to H351. Between H2 and H3 there is a short loop. The first β-strand is located between H1 and H2 (in ABD2 this is I323 to F327), while the remaining β-strands are found after the third helix (in ABD2 S2 = V354 to E357 and an irregular third strand S3 = V370 to L372). S2 and S3 are separated by a loop, which forms the wing. The three strands form an anti-parallel β-sheet. In some winged helix-turn-helix domains a second wing is found after the third β-strand, but this is not present in ABD2. This is not because of any artificially chosen domain boundaries, but is instead due to the full length Ms1 protein ending at residue 375. A second, C-terminal, wing is also missing in other, otherwise typical, winged helix-turn-helix domains such as in histone H5 and E2F4 [35].

### DNA binding

**Fig 2:**
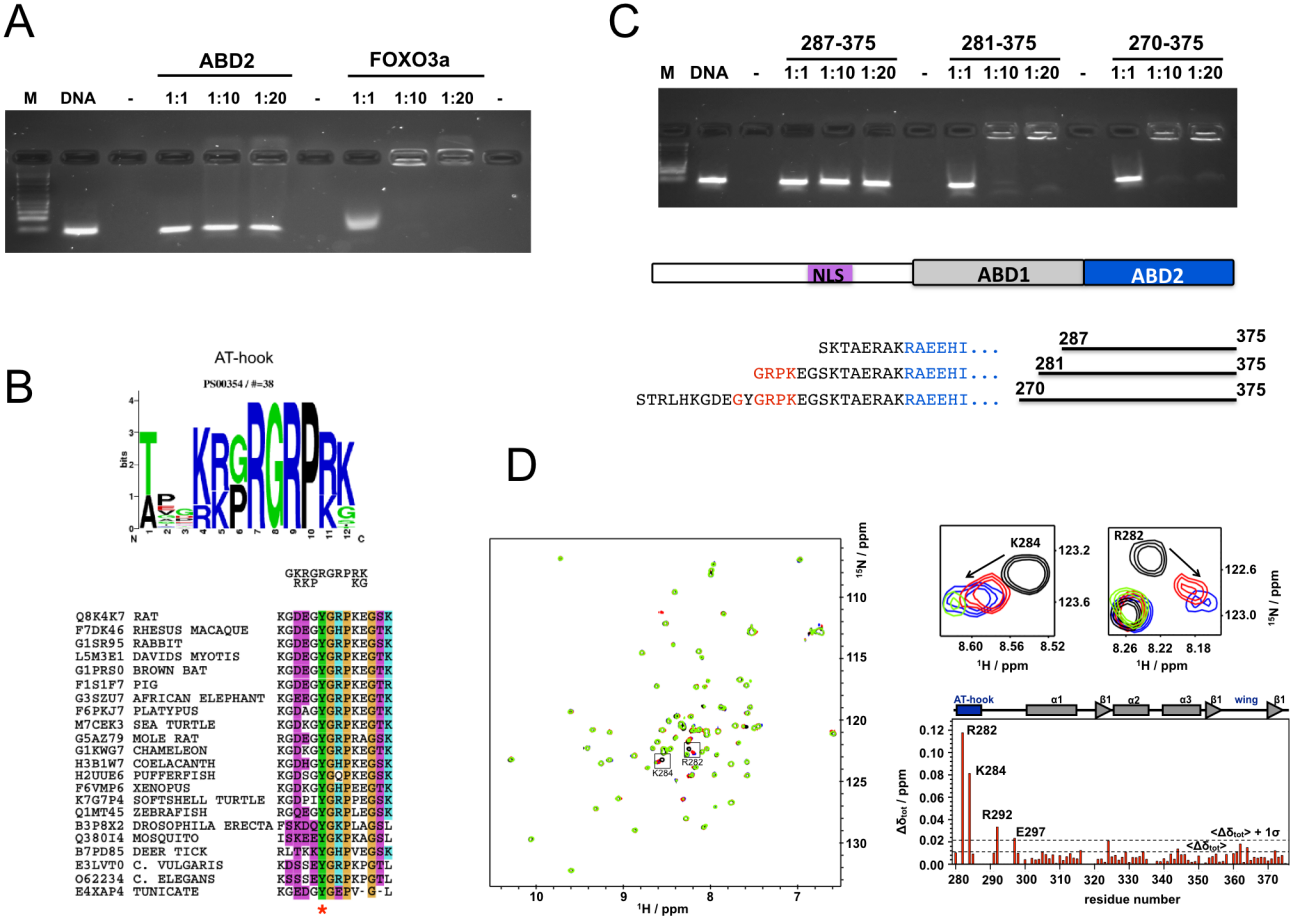
DNA binding of Ms1. A: EMSA experiment of ABD2 and FOXO3a as a positive control with a random 18mer library (2 μM). B: consensus motif of the AT-hook shown as web logo [40] (based on PROSITE entry PDOC00306) and in short form on top of a sequence alignment of the AT-hook motif in selected Ms1 sequences. Colours are: purple—hydrophilic; green—hydrophobic; orange—proline and glycine; blue—positive charge. Phosphorylation sites identified experimentally are indicated by a star [15,41]. C: EMSA of N-terminally extended ABD2 constructs with the same 18mer random library used in A. The overall Ms1 topology is shown together with the detail of the N-terminus of the various constructs. Residues at the N-terminus of ABD2 are shown in blue and those of the AT-hook in red. D: NMR analysis of the interaction of Ms1 270–375 with AT-rich DNA ds oligonucleotides. Spectra of Ms1 270–375 without (black) and with (1:0.5 red; 1:1 blue; 1:2 green) of the oligonucleotide are shown superimposed together with a detailed view for two selected residues from the N-terminus. The chemical shift perturbations are summarised in a plot against the amino acid sequence that is annotated with the functional and structural properties of the 270–375 construct.

Initial DNA binding experiments using electrophoretic mobility shift assays (EMSA) with ABD2 only gave a smear at very high protein concentrations but no clear shifted band, as in the case of the positive control, using a random DNA library in good agreement with results reported for COSTARS [12] (Fig 2A). However, it is not uncommon that monomeric, winged helix loop helix domains are unable to bind to DNA on their own. Instead, it is well documented that they require an additional DNA binding motif, often referred to as “wing 2” [36,37], to bind to DNA. We analysed the sequence N-terminal of the beginning of ABD2 at residue 294 to see if there were any putative DNA binding motifs present that could act as a second wing. We found a well defined AT-hook motif [38] starting from residue 279 in reasonably good agreement with the consensus motif (Fig 2B) which is well conserved in Ms1 throughout evolution from invertebrates to mammals. Three N-terminally extended constructs of ABD2 were generated starting from residues 270, 281 and 287 and used in another round of EMSA experiments (Fig 2C). Strong shifts comparable to the positive control in Fig 2A are seen for constructs starting at residue 281 and 270. The construct starting at residue 287 does not contain the AT-hook and consequently behaves similarly to ABD2. To test if indeed the predicted AT-hook is present and active in DNA binding we tested binding of Ms1 270–375 against double stranded oligonucleotides containing the sequence 5’-CGAATTAATTCG-3’ which is typical for AT-hooks and which was previously shown to bind to the AT-hook of HMGA1 [39]. Significant chemical shift perturbations are indeed observed for the AT-hook alone while the rest of the construct does not show significant changes in the spectrum (Fig 2D).

### DNA binding specificity

**Fig 3:**
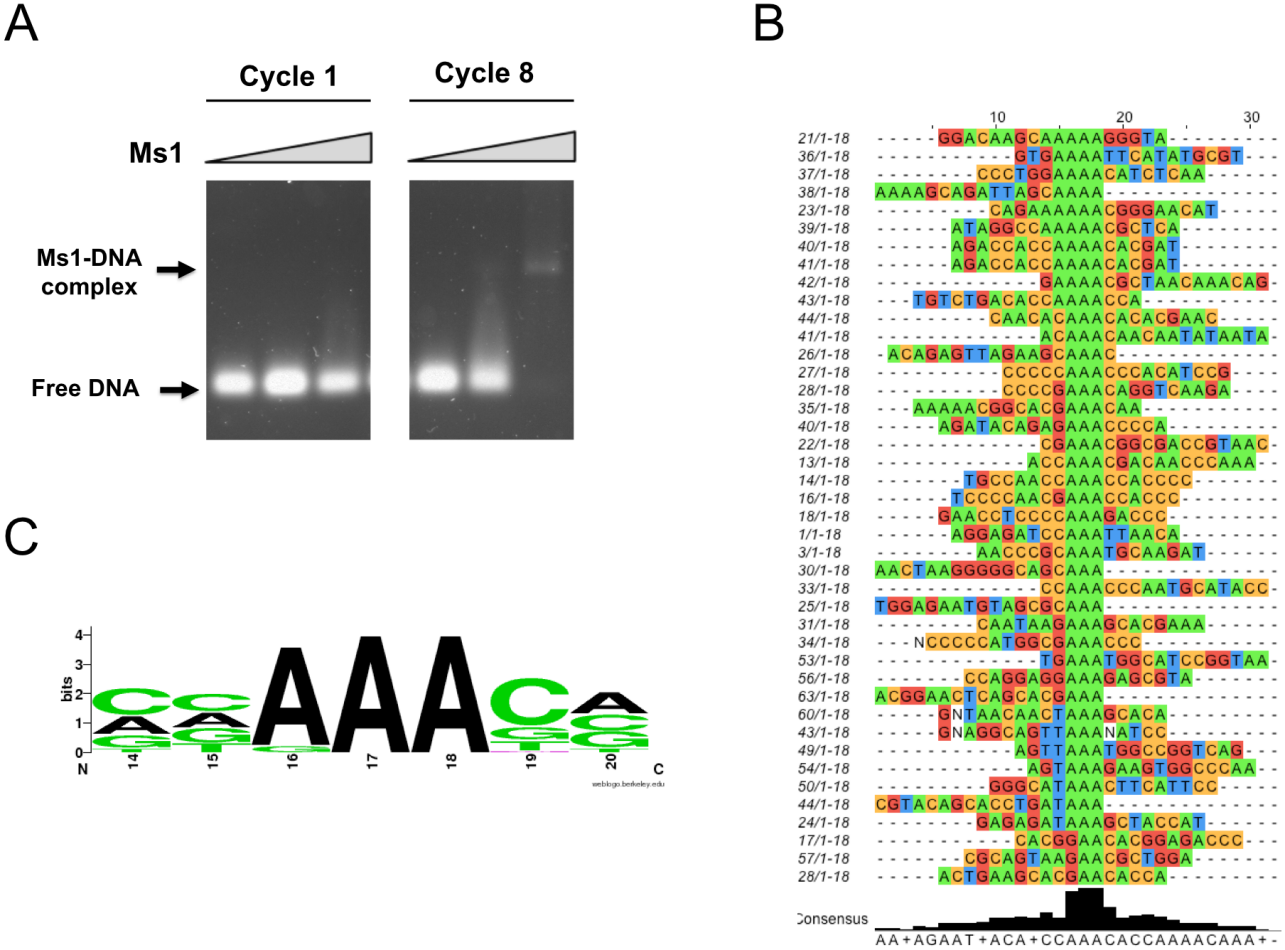
The specificity of DNA binding. A: Gel shift assays with DNA pools from stages 1 (first) and 8 (last) of the SELEX procedure with Ms1 270–375. Positions of free and bound DNA are indicated. B: Sequencing results from the final SELEX pool after stage 8. Only random region shown, constant region is omitted. C: Web logo [40] plot of the consensus DNA recognition motif of ABD2.

**Fig 4:**
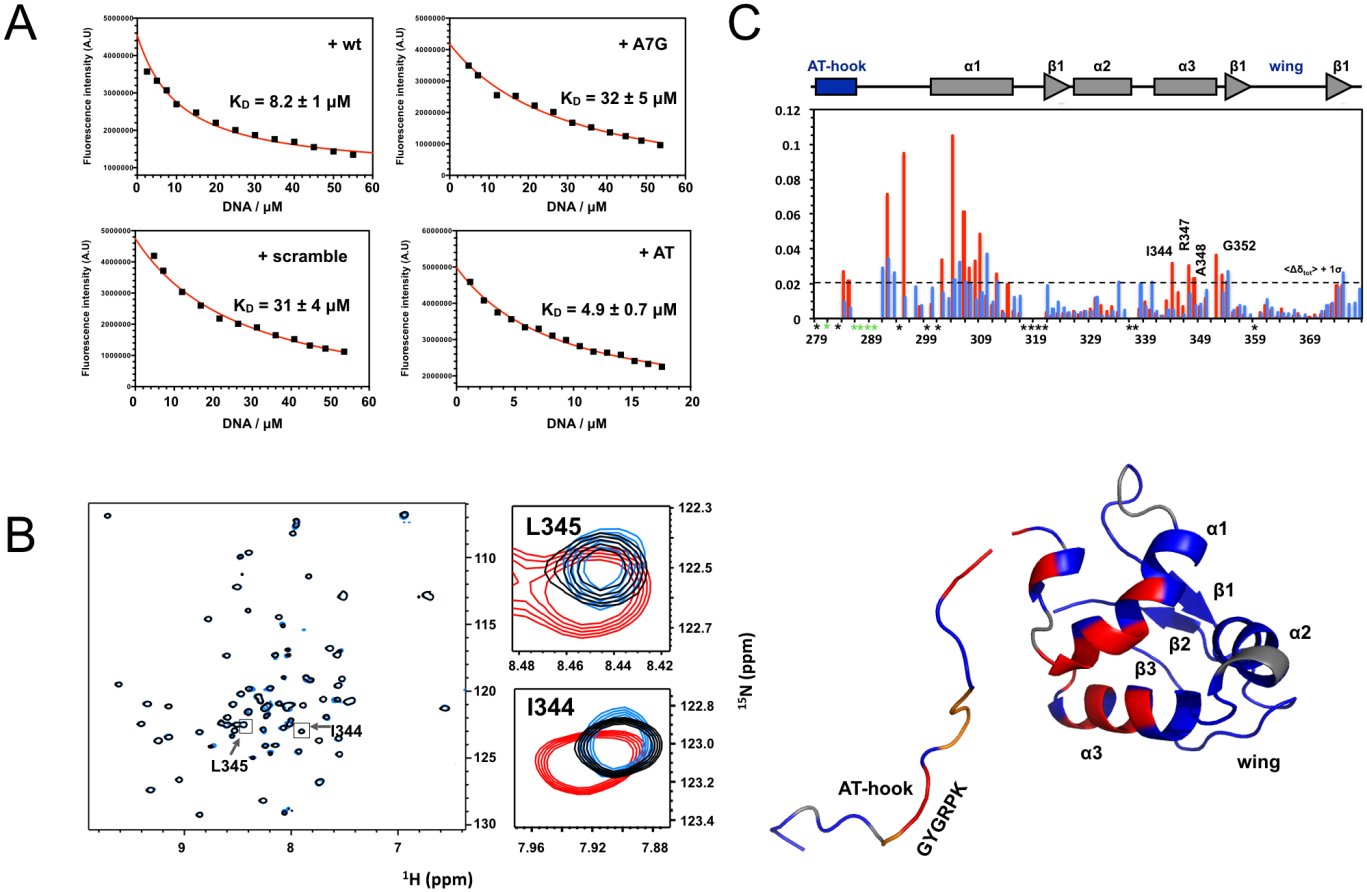
Details of DNA recognition by Ms1. A: Fluorescence titrations of Ms1 270–375 with a double strand oligonucleotide representing the motif identified in the SELEX procedure (wt), a version in which the central A of the motif is replaced by a G (A7G), the scrambled version of wt (scrambled) and a version with additional AT base pairs (AT). Data points are shown in black, the fitting result in red. B: A full view and selected parts of ^15^N-^1^H HSQC spectra of Ms1 270–375 without (black), with the addition of the wt oligo (red) and the A7G mutant oligo (blue) at a 2:1 ratio. Zoomed views are shown for two residues from helix 3. C: plot of chemical shift perturbations of NMR interaction experiments of Ms1 270–375 with the wt (red) and A7G oligo (blue) against the protein sequence. The dotted horizontal line represents the 1*σ noise level. Amino acids for which assignments are missing are labelled with black stars, those that disappear in the titration are labelled with green stars. D: mapping of residues with significant chemical shift perturbations on the surface of the structure of ABD2 (red: chemical sift perturbatios > 1*σ, orange: residues that disappear in the titration, grey: reidues for which assignments are missing). For residues 294–375 our experimental ABD2 structure is used to which residues 270–294 were added based on a homology model manually created using the known structure of an AT-hook (PDB ID 2EZD).

After the identification of the Ms1 construct most suitable to bind to DNA double strands we performed a SELEX experiment to test for sequence specificity. This involved a random library with a random element of 18 bp extended by constant segments for cloning and annealing of PCR primers (Gene Link). Protein to DNA ratio was set such that in the initial round no shifted band was visible in normally stained agarose gels. The SELEX procedure was repeated for 8 cycles whereupon a precise band became clearly visible (Fig 3A). The PCR product after cycle 8 was cloned into pCR 2.1 vector using a TA cloning kit (Invitrogen). A total of 100 positive clones were sequenced and 42 interpretable sequences were obtained (shown in Fig 3B). A good agreement is seen for the conserved sequence of (C/A/G)-AAA-(C/G) shown as web logo [40] in Fig 3C. Synthetic oligonucleotides were synthesised incorporating this motif into the sequence 5’-GACACAAACACAATAG-3’, which also contained an explicit AT rich sequence flanking the conserved motif (wt and +AT oligos) because no clear sequence pattern for an AT-hook binding site could be seen. Fluorescence titrations were performed using the tryptophan residue in the wing of ABD2. Good, saturable binding is observed with an affinity of 4.9 +- 0.7 μM (Fig 4A) for the extended +AT oligo and with an affinity of 8.2 ± 1 μM to the wt oligo. To test the specificity of the core motif, a similar oligonucleotide was used in which the central A of the conserved motif was replaced by a G (A7G oligo). Binding of this mutant was much reduced to an affinity of 32 +- 5 μM and saturation of binding was much less apparent, similar to an oligo that had the sequence of the wt olio mixed (scramble) and bound with an affinity of 31 ± 4 μM.

To ensure that the conserved motif was indeed recognised by helix 3 in ABD2 the interaction of both oligonucleotides with the protein was probed by NMR spectroscopy. It can be seen in Fig 4B–4D that the AT-hook region, the linker that connects it with ABD2, helix 1 and helix 3 show significant chemical shift perturbations with the wt oligo. In contrast, using the A7G oligo the magnitude of the perturbations is globally reduced, reflecting the reduced binding affinity (Fig 4B and 4C). More importantly, a marked dent in the perturbation profile is seen for helix 3 suggesting that indeed helix 3, the recognition helix, does contact the conserved motif in the wt oligo. Mapping the most significant chemical shift perturbations on the structure of ABD2 shows that only the regions facing the DNA respond to binding of the oligonucleotide with the exception of helix 1 and parts of the N-terminus which are further away (Fig 4D).

### Subcellular localisation of Ms1

In previous reports Ms1 was mainly described as a cytosolic protein found attached to stress fibres in non-muscle cells or to thin filaments in the sarcomeres of muscle cells [3]. Occasionally Ms1 was sighted in the nucleus, even though the data were not shown [3] or the observation was not followed up [8]. To avoid confusion of development stages and cell/tissue types we decided to focus on cardiomyocytes, in which the protein was first described, and to only use primary cultures. In that way we could be sure that the cells were fully differentiated containing all potential partner proteins that Ms1 might require to fully function. We used adult rat cardiomyocytes (ARC) as representative of adult tissue and neonatal rat cardiomyocytes (NRC) as a model for development and the stress response in adult tissue.

**Fig 5:**
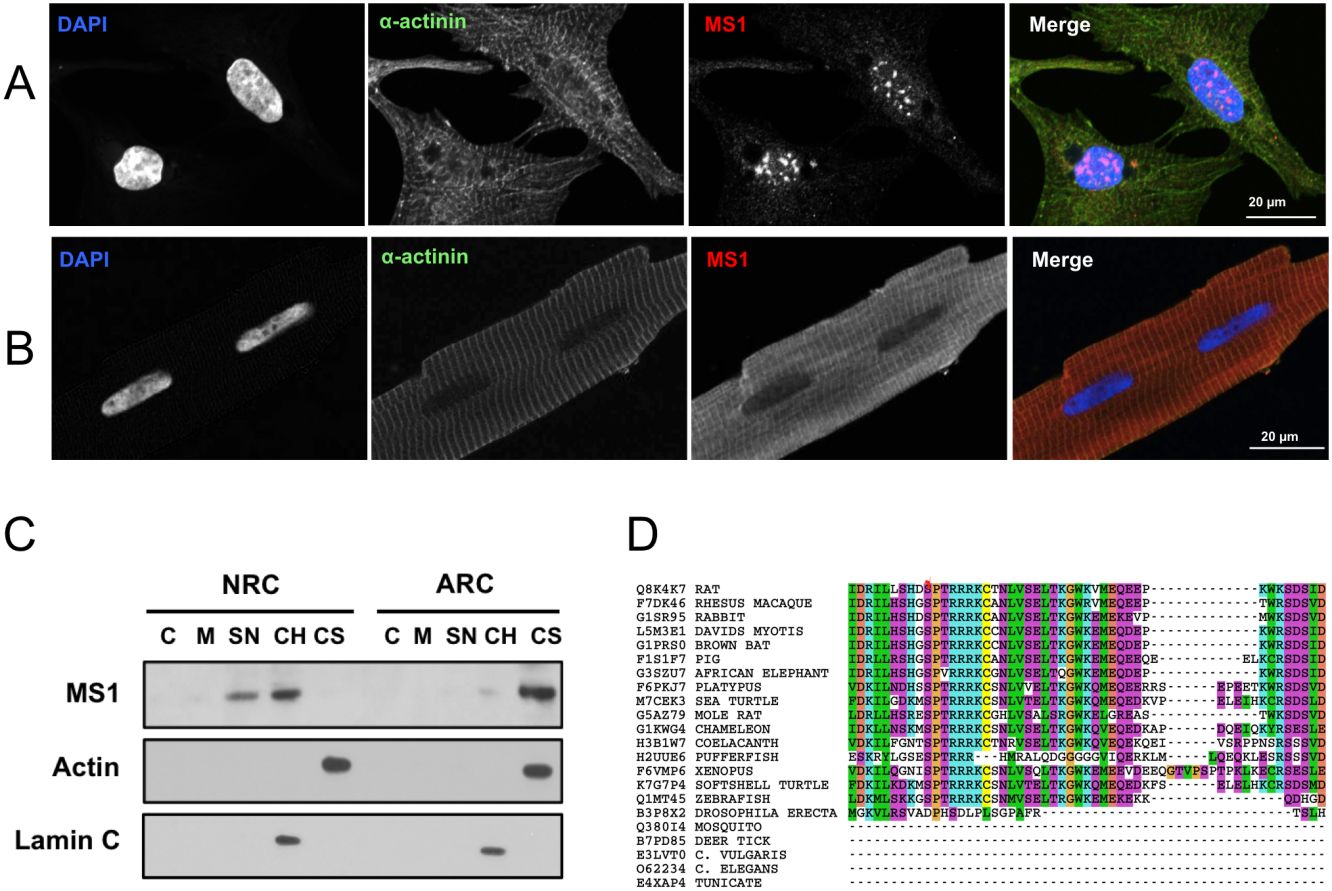
Subcellular localisation of Ms1. A: Immunofluorescent detection of endogenous Ms1 in NRCs. Staining is (left-to-right) DAPI (nuclei, blue), α-actinin (green) and Ms1 (red). B: as A but in ARCs. C: western blot detection of Ms1 in subcellular fractions isolated from ARC and NRC cultures. C = cytoplasm, M = membrane, SN = soluble nuclear, CH = chromatin bound, CS = cytoskeleton. D: Sequence alignment of Ms1 in the region of the nuclear localisation signal (NLS). Phosphorylation sites identified experimentally are indicated by a star [41]. Colours and the subset of sequences are identical to those in Fig 2B. Note that Ms1 from lower organisms lack this region of the protein.

Using a new antibody generated against ABD2 (aABD2chn, S4 and S5 Figs) we were able to detect endogenous Ms1 easily in both NRCs and ARCs using both immunofluorescence and western blots of subcellular fractions. As shown in Fig 5A in NRCs Ms1 is found exclusively in the nucleus where it is stained in a punctate pattern reminiscent of PML bodies [42]. In contrast, in ARCs Ms1 is found exclusively in the sarcomere and the cytosol (Fig 5B). A broadly similar picture emerges from western blots of subcellular fractions of NRCs and ARCs in Fig 5C: In NRCs all of the endogenous Ms1 is found in the nuclear fractions, the majority of which is in the chromatin bound pool. In contrast, endogenous Ms1 in ARCs is found essentially only in the cytosol.

**Fig 6:**
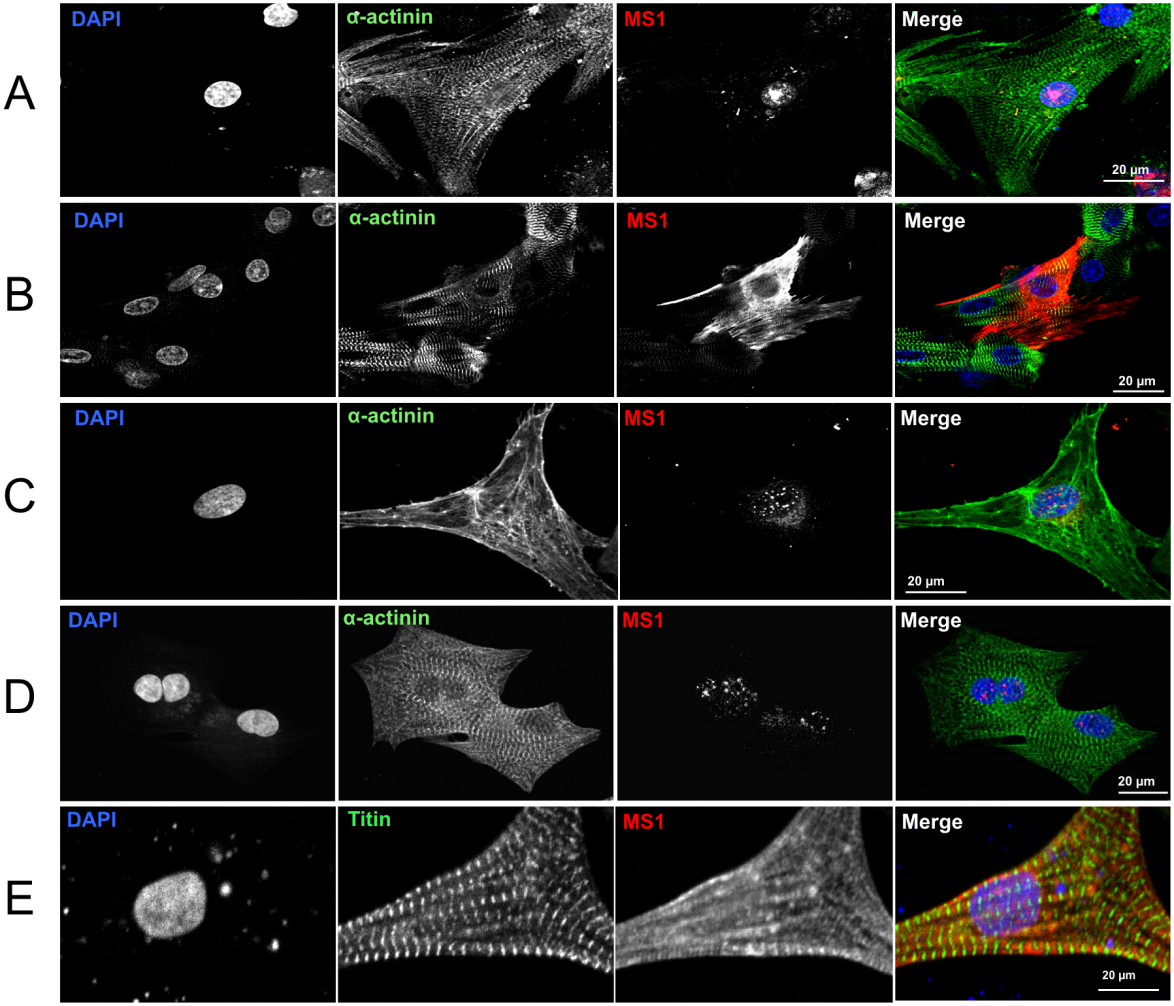
Regulation of the subcellular localisation of Ms1. A: Overexpression of wt myc-tagged Ms1 in culture medium with phenylephrine; detection with anti-myc antibody. B: Overexpression of wt Ms1 in NRCs in culture medium without phenylephrine; detection with anti-myc antibody. C: Detection of endogenous Ms1 with aABD2chn in NRCs grown in culture medium without phenylephrine. D: Detection of endogenous Ms1 with aABD2chn in NRCs grown in culture medium with phenylephrine. E: Overexpression of Ms1 with RR->AA mutation of NLS in NRCs grown in presence of phenylephrine; detection with anti-myc antibody.

To investigate the potential mechanism of nuclear translocation we searched for a nuclear localisation signal (NLS) which we found using PredictNLS [16] starting at residue R153 (RRRKCTNLVSKLTKGWKVMEQEEPKWKS) in rat Ms1. The sequence alignment in Fig 5D shows that this NLS is highly conserved in all vertebrate Ms1 sequences that also contain the AT-hook. As a further confirmation of the detection of Ms1 we overexpressed myc-tagged wild type mouse Ms1 in NRCs. Interestingly, overexpressed Ms1 was found in the nucleus only in hypertrophic medium (Fig 6A) while it was in the cytosol in non hypertrophic medium. (Fig 6B). In contrast, endogenous Ms1 was found in the nucleus of NRCs regardless of the type of medium (Fig 6A: non hypertrophic medium & Fig 6B: hypertrophic medium).

To establish if the nuclear translocation was dependent on the NLS shown in Fig 5D we mutated two of the four positively charged residues in the core of the NLS of Ms1 to alanine (RR->AA). Overexpression of this mutant, even in the presence of phenylephrine, abolished nuclear translocation (Fig 6E).

## Discussion

The structure determination of the only folded domain in the cardiac stress response factor Ms1 gave the unexpected result of looking more like the DNA binding domain of a transcription factor than an actin binding protein. A very similar result was obtained for the smaller version of Ms1, COSTARS [12] even though this protein neither bound DNA nor actin. The structures are overall very similar even though some significant local structural differences were observed, most notable in the portion between helix 1 and strand 1 where COSTARS contains a short extra strand whereas Ms1 ABD2 has a short helix (Fig 1C). Inspection of the raw NMR data suggests that these divergent structures are indeed genuine and not artefacts, possibly reflecting the rather distinct sequences of both proteins in this part of the structure (the highly positively charged sequence ARHRR in Ms1 compared to GSKNA in COSTARS).

In agreement with data reported for COSTARS, Ms1 ABD2 did not bind clearly to DNA on its own. However, analysis of the immediately preceding sequence of Ms1 revealed the presence of an AT-hook, which was validated experimentally using NMR spectroscopy. A closer inspection of the AT-hook sequence in Ms1 shows that while the core GRP motif is well conserved throughout (Fig 2B), several basic flanking amino acids are replaced by neutral or even negatively charged amino acids. Such a variation of this motif would be expected to significantly weaken the binding affinity of the AT-hook compared to more ideal versions. It is assumed that this could serve the purpose of ensuring that significant DNA binding occurs only in cooperation of both motifs, the AT-hook and ABD2. A weakly binding AT-hook could also aid scanning of DNA by one dimensional diffusion until a specific site is encountered [43,44]. Ms1 Constructs containing both AT-hook and ABD2 produced shifts in EMSA experiments comparable to the positive control FOXO3a (Fig 2A and 2C). Little effect is seen at a DNA:protein ratio of 1:1 while almost all DNA is in a complex at 1:10 and 1:20. Such a behaviour is in good agreement with the assumption of a K_D_ value on the order of magnitude of tens of μM (comparable to unspecific binding of Ms1 to the scramble or A7G oligonucleotides) so that less than 5% of complex would be formed at a ratio of 1:1 but more than 70% at a ratio of 1:10 and more than 85% at a ratio of 1:20.

The SELEX experiment produced a well defined DNA motif that was validated using synthetic oligonucleotides. Fluorescence titrations showed a low micro molar binding affinity for the wild type sequence. While numerous transcription factors bind to their recognition sites with nanomolar affinities [45,46] others do bind with affinities comparable to that of ABD2, e.g. AsH2L with a K_D_ of about 4 μM [47] or various CXXC domains with K_D_ values from 0.6 to 22 μM [48]. A single nucleotide mutation in the centre of the motif reduced the binding affinity several fold as did an oligonucleotide with a scrambled sequence. Specific binding is also confirmed by NMR titration experiments that show significant chemical shift perturbations for the AT-hook and recognition helix 3 which are almost completely abolished in titrations with the A7G mutant oligonucleotide. Curiously, also the linker between the AT-hook and parts of helix 1 show even larger chemical shift perturbations albeit no direct interaction with DNA is expected in this part of the protein. However, as the N-terminus of Ms1 is opposite the DNA recognition helix it follows that the linker sequence has to run past the rest of the domain to allow the AT-hook to approach the DNA. Consequently, the linker residues would come close to the N-terminus of ABD2 and could even make stabilising contacts. This is supported by the observation of increasing melting temperatures for the increased length of the N-terminally extended constructs (S6 Fig). No changes in melting temperature would be expected in the absence of interactions. Such interactions are also directly observed in comparisons of chemical shift differences of shorter and longer constructs which show chemical shift perturbations in helix 1 similar to those observed upon DNA binding (S6 Fig). This part of the protein will also experience significant conformational changes upon DNA binding which explains the disproportionately large chemical shift perturbations in comparison to the recognition helix where they are more moderate. The latter suggests that the recognition helix does not change conformation very much and that even the side chains need only small changes to fit into the major groove of the DNA molecule.

To act as a transcription factor a protein ought to be able to reach the nucleus. A standard means to get there is a nuclear localisation signal (NLS), which does indeed exist in Ms1 following residue R153. Interestingly, though, so far most reports in the literature reported essentially exclusively cytoplasmic localisation. Occasional sightings [3,8] were not followed up to our knowledge. We found Ms1 in the nucleus so far only under what appears to be tightly controlled conditions in primary cultures of neonatal rat cardiomyocytes (NRCs). Both subcellular fractionation and immunofluorescence suggest that Ms1 fulfils a specific function in the nucleus: by showing that it is tightly associated with the chromosomal material (fractionation) and by its appearance in a speckled or spotted pattern in the nucleus (immunofluorescence). Such patterns, e.g. PML bodies [42,49], are thought to correspond to increased activity of DNA replication, splicing or transcription. It remains to be seen into which of these categories of function Ms1 falls. Interestingly, while endogenous Ms1 was always in the nucleus in NRCs, overexpressed Ms1 was only in the nucleus in hypertrophic medium. This suggests a complex pattern of spatiotemporal regulation of Ms1 where an early developmental signal is expected to drive Ms1 into the nucleus, which is already switched off at birth. As a consequence, overexpressed Ms1 requires extra signals to get into the nucleus. Due to a lack of a nuclear export signal (based on sequence analysis of rat Ms1 with NetNES 1.1 server [50]) nuclear export of Ms1 must be less efficient, in good agreement with our observations. Therefore, it can be assumed that Ms1 is actively transported to the nucleus early in cardiac development, a pattern that is expected to be repeated in the stress response of adult cardiomyocytes. At the latest around birth this nuclear import is switched off but is not accompanied by active export of Ms1. Nevertheless at some point in postnatal development nuclear export must happen because in ARCs Ms1 is not in the nucleus anymore. Our data suggest that nuclear import depends on the NLS as its mutation abolished it. Interestingly, it has been shown experimentally that S156 (human numbering, S150 in mouse and rat, Fig 5D) is phosphorylated by a p38 like kinase [15,41] which could regulate its activity [51,52]. Other opportunities for regulation of Ms1 activity exist with regards to DNA binding where the conserved Y286 in the AT-hook and S338 at the N-terminus of the recognition helix have been shown experimentally to be phosphorylated [15,41].

In conclusion we have shown that Ms1 possesses all the necessary attributes to be directly involved in control of transcription: it can bind to specific sites in DNA and can translocate to the nucleus. While it is too early to make specific predictions as to its precise function in the nucleus it might be fruitful to speculate as to what it might be. We would therefore like to suggest that Ms1 in the nucleus is involved in the stimulation of the expression of genes needed for the buildup of muscle mass as required both in early embryonal development as well as in the hypertrophic response to stress situations in adults. Ms1 could do so most likely directly as a transcription factor or less likely indirectly via an involvement in mRNA processing. Such a function is supported by the assumption that its nuclear translocation is driven by upstream signals acting via a p38-like kinase which in turn would be activated by hypertrophic signalling. Having a well defined postulated function will facilitate further experimental exploration of this unexpectedly versatile stress response protein.

## Supporting Information

S1 Fig Alignment of a selection of full length Ms1 protein sequences. A selection of functional features, structural domains and secondary structure elements are shown by bars. Experimentally identified phosphorylation sites that are conserved amongst mammals are indicated by red stars.
(PDF)

S2 Fig Backbone dynamics analysis of ABD2. On the left are shown the experimental values for ^15^N T_1_, T_2_ and heteronuclear NOE recorded at 500 MHz. On the right are two parameters from the Lipari-Szabo analysis, the order parameter S^2^ and the exchange contribution R_ex_. Apart from a small region in the wing there is little flexibility in the compact structure.
(PDF)

S3 Fig Top 50 hits of DALI search of the PDB with the structure of ABD2. Proteins that are not involved in DNA binding are marked with a blue star. Structures used in the superposition in Fig 1D are marked with red stars.
(PDF)

S4 Fig Western blot validation of new antibody against Ms1. The antibody (aABD2chn) was generated for detection of endogenous Ms1. It was validated by western blots with myc-tagged full length Ms1 expressed in COS cells (left); western blots of endogenous Ms1 in muscle tissue extracts with untreated and blocked antibody (lower left); western blots of overexpressed myc-tagged full length Ms1 in HeLa cells (below);
(PDF)

S5 Fig Immunofluorescence validation of new antibody Ms1. **I**mmunofluorescence detection of transfected full length myc-tagged Ms1 in HeLa cells using anti-myc as well as aABD2chn antibodies (left) and immunofluorescence detection of endogenous Ms1 in NRCs with untreated and blocked aABD2chn (right).
(PDF)

S6 Fig Effects of potential interactions of the N-terminal extension of ABD2 on stability and NMR spectra of ABD2. A significant increase in stability of > 7°C is seen for the addition of 23 amino acids at the N-terminus of ABD2.This addition leads to considerable chemical shift changes around helix one to which it would need to pack to allow the AT-hook to reach the DNA.
(PDF)

## References

[1] Wallace MA, Lamon S, Russell AP. The regulation and function of the striated muscle activator of rho signaling (STARS) protein. Front Physiol. 2012;3:469 10.3389/fphys.2012.00469 23248604 PMC3520124

[2] Mahadeva H, Brooks G, Lodwick D, Chong NW, Samani NJ. ms1, a novel stress-responsive, muscle-specific gene that is up-regulated in the early stages of pressure overload-induced left ventricular hypertrophy. FEBS Lett. 2002 6 19;521(1–3):100–4. 12067735 10.1016/s0014-5793(02)02833-8

[3] Arai A, Spencer JA, Olson EN. STARS, a Striated Muscle Activator of Rho Signalling and Serum Response Factor-dependent Transcription. JBiolChem. 2002;277:24453–9. 10.1074/jbc.M202216200 11983702

[4] Chong NW, Koekemoer AL, Ounzain S, Samani NJ, Shin JT, Shaw SY. STARS Is Essential to Maintain Cardiac Development and Function In Vivo via a SRF Pathway. PLOS ONE. 2012;7(7):e40966 10.1371/journal.pone.0040966 22815879 PMC3399798

[5] Kuwahara K, Teg Pipes GC, McAnally J, Richardson JA, Hill JA, Bassel-Duby R, et al Modulation of adverse cardiac remodeling by STARS, a mediator of MEF2 signaling and SRF activity. JClinInvest. 2007 5;117(5):1324–34. 10.1172/JCI31240 PMC1838928 17415416

[6] Ounzain S, Kobayashi S, Peterson RE, He A, Motterle A, Samani NJ, et al Cardiac Expression of ms1/STARS, a Novel Gene Involved in Cardiac Development and Disease, Is Regulated by GATA4. MolCellBiol. American Society for Microbiology; 2012 5 15;32(10):1830–43. 10.1128/MCB.06374-11 PMC3347400 22431517

[7] Koekemoer AL, Chong NW, Goodall AH, Samani NJ. Myocyte stress 1 plays an important role in cellular hypertrophy and protection against apoptosis. FEBS Lett. 2009 9 3;583(17):2964–7. 10.1016/j.febslet.2009.08.011 19686740

[8] Troidl K, Rüding I, Cai W-J, Mücke Y, Grossekettler L, Piotrowska I, et al Actin-binding rho activating protein (Abra) is essential for fluid shear stress-induced arteriogenesis. Arterioscler Thromb Vasc Biol. 2009 12;29(12):2093–101. 10.1161/ATVBAHA.109.195305 19778941

[9] Pang T-L, Chen F-C, Weng Y-L, Liao H-C, Yi Y-H, Ho C-L, et al Costars, a Dictyostelium protein similar to the C-terminal domain of STARS, regulates the actin cytoskeleton and motility. JCellSci. 2010 11 1;123(Pt 21):3745–55. 10.1242/jcs.064709 20940261

[10] Fogl C, Puckey L, Hinssen U, Zaleska M, El-Mezgueldi M, Croasdale R, et al A structural and functional dissection of the cardiac stress response factor Ms1. Proteins. 2011 9 19. 10.1002/prot.23201 22081479

[11] Barrientos T, Frank D, Kuwahara K, Bezprozvannaya S, Pipes GCT, Bassel-Duby R, et al Two novel members of the ABLIM protein family, ABLIM-2 and -3, associate with STARS and directly bind F-actin. J Biol Chem. 2007 3 16;282(11):8393–403. 17194709 10.1074/jbc.M607549200

[12] Lin J, Zhou T, Wang J. Solution structure of the human HSPC280 protein. Protein Sci. 2011 1;20(1):216–23. 10.1002/pro.548 21082705 PMC3047078

[13] Schröder S, Fraternali F, Quan X, Scott D, Qian F, Pfuhl M. When a module is not a domain: the case of the REJ module and the redefinition of the architecture of polycystin-1. BiochemJ. 2011 5 1;435(3):651–60. 21314639 10.1042/BJ20101810 PMC4979573

[14] Lee T-Y, Bo-Kai Hsu J, Chang W-C, Huang H-D. RegPhos: a system to explore the protein kinase-substrate phosphorylation network in humans. Nucleic Acids Res. 2011 1;39(Database issue):D777–87. 10.1093/nar/gkq970 21037261 PMC3013804

[15] Hornbeck PV, Kornhauser JM, Tkachev S, Zhang B, Skrzypek E, Murray B, et al PhosphoSitePlus: a comprehensive resource for investigating the structure and function of experimentally determined post-translational modifications in man and mouse. Nucleic Acids Res. 2012 1;40(Database issue):D261–70. 10.1093/nar/gkr1122 22135298 PMC3245126

[16] Cokol M, Nair R, Rost B. Finding nuclear localization signals. EMBO Rep. 2000 11;1(5):411–5. 11258480 10.1093/embo-reports/kvd092 PMC1083765

[17] Vranken WF, Boucher W, Stevens TJ, Fogh RH, Pajon A, Llinas M, et al The CCPN data model for NMR spectroscopy: development of a software pipeline. Proteins. 2005 6 1;59(4):687–96. 15815974 10.1002/prot.20449

[18] Delaglio F, Gzesiek S, Vuister GW, Zhu G, Pfeifer J, Bax A. NMRPipe: A multidimensional spectral processing system based on UNIX pipes. JBiomolNMR. 1995;6:277–93. 10.1007/BF00197809 8520220

[19] Guntert P, Mumenthaler C, Wüthrich K. Torsion angle dynamics for NMR structure calculation with the new program DYANA. Journal of Molecular Biology. ENGLAND; 1997;273(1):283–98. Available: http://www.sciencedirect.com/science/article/pii/S0022283697912845 10.1006/jmbi.1997.1284 9367762

[20] Holm L, Sander C. Protein structure comparison by alignment of distance matrices. Journal of Molecular Biology. ENGLAND; 1993;233(1):123–38. Available: http://www.sciencedirect.com/science/article/pii/S0022283683714890 10.1006/jmbi.1993.1489 8377180

[21] Holm L, Sander C. Dali: a network tool for protein structure comparison. Trends BiochemSci. ENGLAND; 1995;20(11):478–80. Available: http://www.sciencedirect.com/science/article/pii/S0968000400891057 10.1016/s0968-0004(00)89105-7 8578593

[22] Tsai K-L, Sun Y-J, Huang C-Y, Yang J-Y, Hung M-C, Hsiao C-D. Crystal structure of the human FOXO3a-DBD/DNA complex suggests the effects of post-translational modification. Nucleic Acids Res. 2007;35(20):6984–94. 17940099 10.1093/nar/gkm703 PMC2175300

[23] Bianchi A, Stansel RM, Fairall L, Griffith JD, Rhodes D, de Lange T. TRF1 binds a bipartite telomeric site with extreme spatial flexibility. EMBO J. ENGLAND; 1999;18(20):5735–44. Available: http://www.nature.com/emboj/journal/v18/n20/abs/7591978a.html 10.1093/emboj/18.20.5735 PMC1171640 10523316

[24] Bailey TL, Boden M, Buske FA, Frith M, Grant CE, Clementi L, et al MEME SUITE: tools for motif discovery and searching. Nucleic Acids Res. 2009 7;37(Web Server issue):W202–8. Available: http://nar.oxfordjournals.org/content/37/suppl_2/W202.long 10.1093/nar/gkp335 19458158 PMC2703892

[25] Zhang M, Kho AL, Anilkumar N, Chibber R, Pagano PJ, Shah AM, et al Glycated proteins stimulate reactive oxygen species production in cardiac myocytes: involvement of Nox2 (gp91phox)-containing NADPH oxidase. Circulation. 2006 3 7;113(9):1235–43. 16505175 10.1161/CIRCULATIONAHA.105.581397

[26] Iskratsch T, Reijntjes S, Dwyer J, Toselli P, Dégano IR, Dominguez I, et al Two distinct phosphorylation events govern the function of muscle FHOD3. Cell Mol Life Sci. 2013 3;70(5):893–908. 10.1007/s00018-012-1154-7 23052206 PMC3696992

[27] Zhang X, Wang W, Bedigian AV, Coughlin ML, Mitchison TJ, Eggert US. Dopamine receptor D3 regulates endocytic sorting by a Prazosin-sensitive interaction with the coatomer COPI. ProcNatlAcadSciUSA. National Acad Sciences; 2012 7 31;109(31):12485–90. 10.1073/pnas.1207821109 PMC3411939 22802617

[28] Iskratsch T, Lange S, Dwyer J, Kho AL, Remedios dos C, Ehler E. Formin follows function: a muscle-specific isoform of FHOD3 is regulated by CK2 phosphorylation and promotes myofibril maintenance. JCell Biol. 2010 12 13;191(6):1159–72. 21149568 10.1083/jcb.201005060 PMC3002041

[29] Barbato G, Ikura M, Kay LE, Pastor RW, Bax A. Backbone dynamics of calmodulin studied by 15N relaxation using inverse detected two-dimensional NMR spectroscopy: the central helix is flexible. Biochemistry (NY). 1992 6 16;31(23):5269–78. 10.1021/bi00138a005 1606151

[30] McKay DB, Steitz TA. Structure of catabolite gene activator protein at 2.9 A resolution suggests binding to left-handed B-DNA. Nature. 1981 4 30;290(5809):744–9. 6261152 10.1038/290744a0

[31] Anderson WF, Ohlendorf DH, Takeda Y, Matthews BW. Structure of the cro repressor from bacteriophage lambda and its interaction with DNA. Nature. 1981 4 30;290(5809):754–8. 6452580 10.1038/290754a0

[32] Clark KL, Halay ED, Lai E, Burley SK. Co-crystal structure of the HNF-3/fork head DNA-recognition motif resembles histone H5. Nature. 1993 7 29;364(6436):412–20. 8332212 10.1038/364412a0

[33] Lai E, Clark KL, Burley SK, Darnell JE. Hepatocyte nuclear factor 3/fork head or “winged helix” proteins: a family of transcription factors of diverse biologic function. ProcNatlAcadSciUSA. 1993 11 15;90(22):10421–3. 10.1073/pnas.90.22.10421 PMC47788 8248124

[34] Gajiwala KS, Burley SK. Winged helix proteins. CurrOpinStructBiol. 2000 2;10(1):110–6. 10.1016/s0959-440x(99)00057-3 10679470

[35] Zheng N, Fraenkel E, Pabo CO, Pavletich NP. Structural basis of DNA recognition by the heterodimeric cell cycle transcription factor E2F-DP. Genes Dev. 1999 3 15;13(6):666–74. 10090723 10.1101/gad.13.6.666 PMC316551

[36] Murphy TC, Saleem RA, Footz T, Ritch R, McGillivray B, Walter MA. The wing 2 region of the FOXC1 forkhead domain is necessary for normal DNA-binding and transactivation functions. Invest Ophthalmol Vis Sci. 2004 8;45(8):2531–8. 15277473 10.1167/iovs.04-0167

[37] Boura E, Silhan J, Herman P, Vecer J, Sulc M, Teisinger J, et al Both the N-terminal loop and wing W2 of the forkhead domain of transcription factor Foxo4 are important for DNA binding. J Biol Chem. 2007 3 16;282(11):8265–75. 17244620 10.1074/jbc.M605682200

[38] Reeves R, Nissen MS. The A.T-DNA-binding domain of mammalian high mobility group I chromosomal proteins. A novel peptide motif for recognizing DNA structure. JBiolChem. 1990 5 25;265(15):8573–82. 1692833

[39] Fonfría-Subirós E, Acosta-Reyes F, Saperas N, Pous J, Subirana JA, Campos JL. Crystal structure of a complex of DNA with one AT-hook of HMGA1. PLOS ONE. 2012;7(5):e37120 10.1371/journal.pone.0037120 22615915 PMC3353895

[40] Crooks GE, Hon G, Chandonia J-M, Brenner SE. WebLogo: a sequence logo generator. Genome Res. 2004 6;14(6):1188–90. 15173120 10.1101/gr.849004 PMC419797

[41] Huttlin EL, Jedrychowski MP, Elias JE, Goswami T, Rad R, Beausoleil SA, et al A tissue-specific atlas of mouse protein phosphorylation and expression. Cell. 2010 12 23;143(7):1174–89. 10.1016/j.cell.2010.12.001 21183079 PMC3035969

[42] Luciani JJ, Depetris D, Usson Y, Metzler-Guillemain C, Mignon-Ravix C, Mitchell MJ, et al PML nuclear bodies are highly organised DNA-protein structures with a function in heterochromatin remodelling at the G2 phase. JCellSci. 2006 6 15;119(Pt 12):2518–31. 10.1242/jcs.02965 16735446

[43] Riggs AD, Bourgeois S, Cohn M. The lac repressor-operator interaction. 3. Kinetic studies. Journal of Molecular Biology. 1970 11 14;53(3):401–17. 4924006 10.1016/0022-2836(70)90074-4

[44] Berg OG, Ehrenberg M. Association kinetics with coupled three- and one-dimensional diffusion. Chain-length dependence of the association rate of specific DNA sites. BiophysChem. 1982 4;15(1):41–51. 10.1016/0301-4622(82)87015-4 7074207

[45] Vacha P, Zuskova I, Bumba L, Herman P, Vecer J, Obsilova V, et al Detailed kinetic analysis of the interaction between the FOXO4-DNA-binding domain and DNA. BiophysChem. 2013 12;184(1):68–78 10.1016/j.bpc.2013.09.002 24121535

[46] Pham TH, Minderjahn J, Schmidl C, Hoffmeister H, Schmidhofer S, Chen W, et al Mechanisms of in vivo binding site selection of the hematopoietic master transcription factor PU.1. Nucleic Acids Res. 2013 7;41(13):6391–6402 10.1093/nar/gkt355 23658224 PMC3711439

[47] Sarvan S, Avdic V, Tremblay V, Chaturvedi CP, Zhang P, Lanouette S, et al Crystal structure of the trithorax group protein ASH2L reveals a forkhead-like DNA binding domain. NatStructMolBiol. 2011 7;18(7):857–859 10.1038/nsmb.2093 PMC3983046 21642971

[48] Risner LE, Kuntimaddi A, Lokken AA, Achille NJ, Birch NW, Schoenfelt K, et al Functional specificity of CpG DNA-binding CXXC domains in mixed lineage leukemia. J Biol Chem. 2013 10;288(41):29901–29910 10.1074/jbc.M113.474858 23990460 PMC3795288

[49] Lallemand-Breitenbach V, de Thé H. PML nuclear bodies. Cold Spring Harb Perspect Biol. 2010 5;2(5):a000661 10.1101/cshperspect.a000661 20452955 PMC2857171

[50] la Cour T, Kiemer L, Mølgaard A, Gupta R, Skriver K, Brunak S. Analysis and prediction of leucine-rich nuclear export signals. Protein EngDesSel. 2004 6;17(6):527–36. 10.1093/protein/gzh062 15314210

[51] Jans DA, Hübner S. Regulation of protein transport to the nucleus: central role of phosphorylation. PhysiolRev. 1996 7;76(3):651–85. Available: http://physrev.physiology.org/content/76/3/651.abstract 10.1152/physrev.1996.76.3.651 8757785

[52] Harreman MT, Kline TM, Milford HG, Harben MB, Hodel AE, Corbett AH. Regulation of Nuclear Import by Phosphorylation Adjacent to Nuclear Localization Signals. J Biol Chem. 2004 5 7;279(20):20613–21. 14998990 10.1074/jbc.M401720200

