# Supplementary material for: The Cardiac Stress Response Factor Ms1 Can Bind to DNA and Has a Function in the Nucleus
Source: PLoS One. 2015 Dec 14;10(12):e0144614. doi: 10.1371/journal.pone.0144614 (PMC4682817; doi:10.1371/journal.pone.0144614)

Alignment of a selection of full length Ms1 protein sequences. A selection of functional features, structural domainsand secondary structure elements are shown by bars. Experimentally identified phosphorylation sites that are conserved amongst mammals are indicated by red stars.

S1 Fig

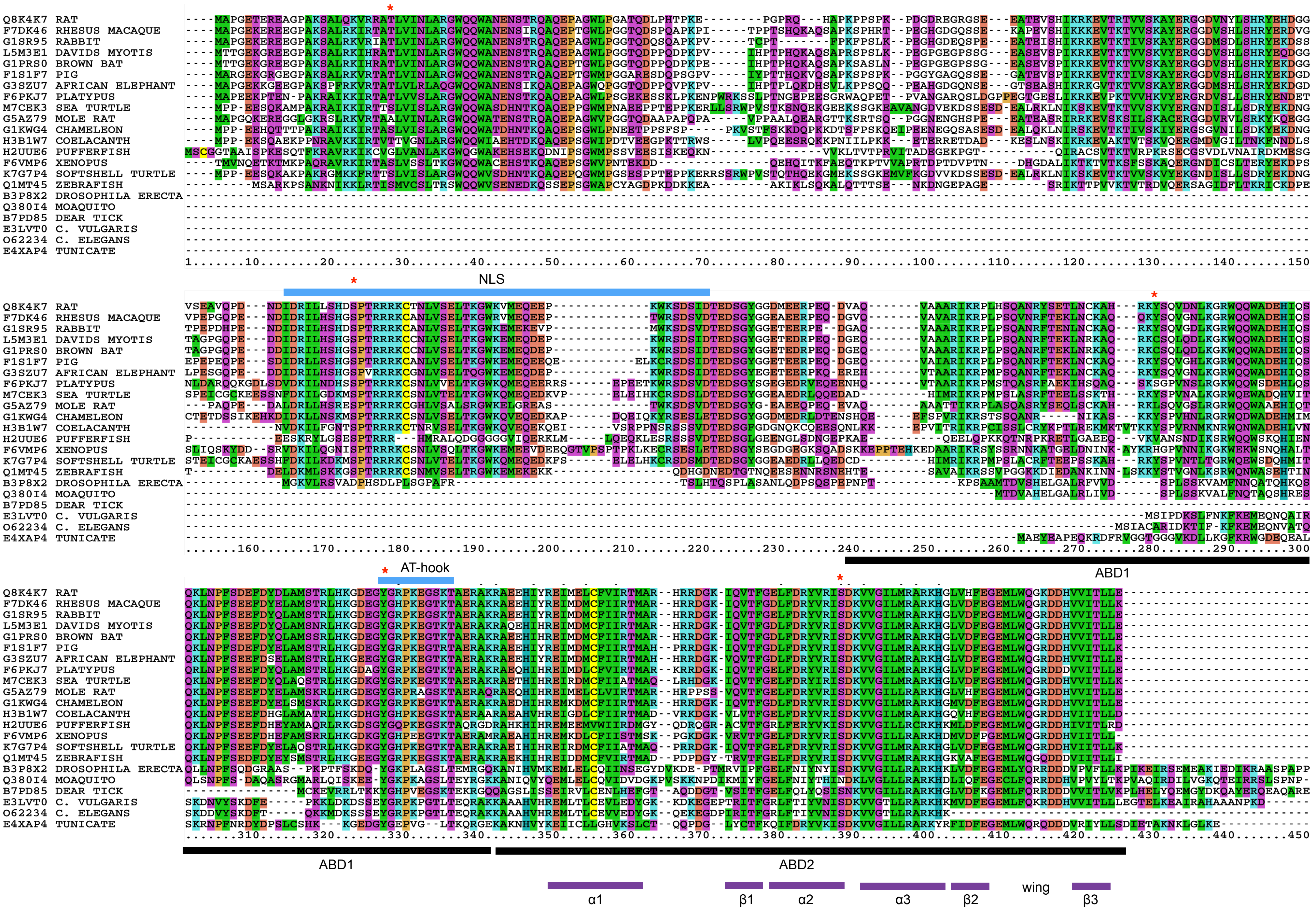

Supplement: S1 Fig — A selection of functional features, structural domains and secondary structure elements are shown by bars. Experimentally identified phosphorylation sites that are conserved amongst mammals are indicated by red stars. (PDF) [file pone.0144614.s001.pdf]
