# Supplementary material for: The Cardiac Stress Response Factor Ms1 Can Bind to DNA and Has a Function in the Nucleus
Source: PLoS One. 2015 Dec 14;10(12):e0144614. doi: 10.1371/journal.pone.0144614 (PMC4682817; doi:10.1371/journal.pone.0144614)
Supplement: S2 Fig — On the left are shown the experimental values for 15N T1, T2 and heteronuclear NOE recorded at 500 MHz. On the right are two parameters from the Lipari-Szabo analysis, the order parameter S2 and the exchange contribution Rex. Apart from a small region in the wing there is little flexibility in the compact structure. (PDF) [file pone.0144614.s002.pdf]

S2 Fig

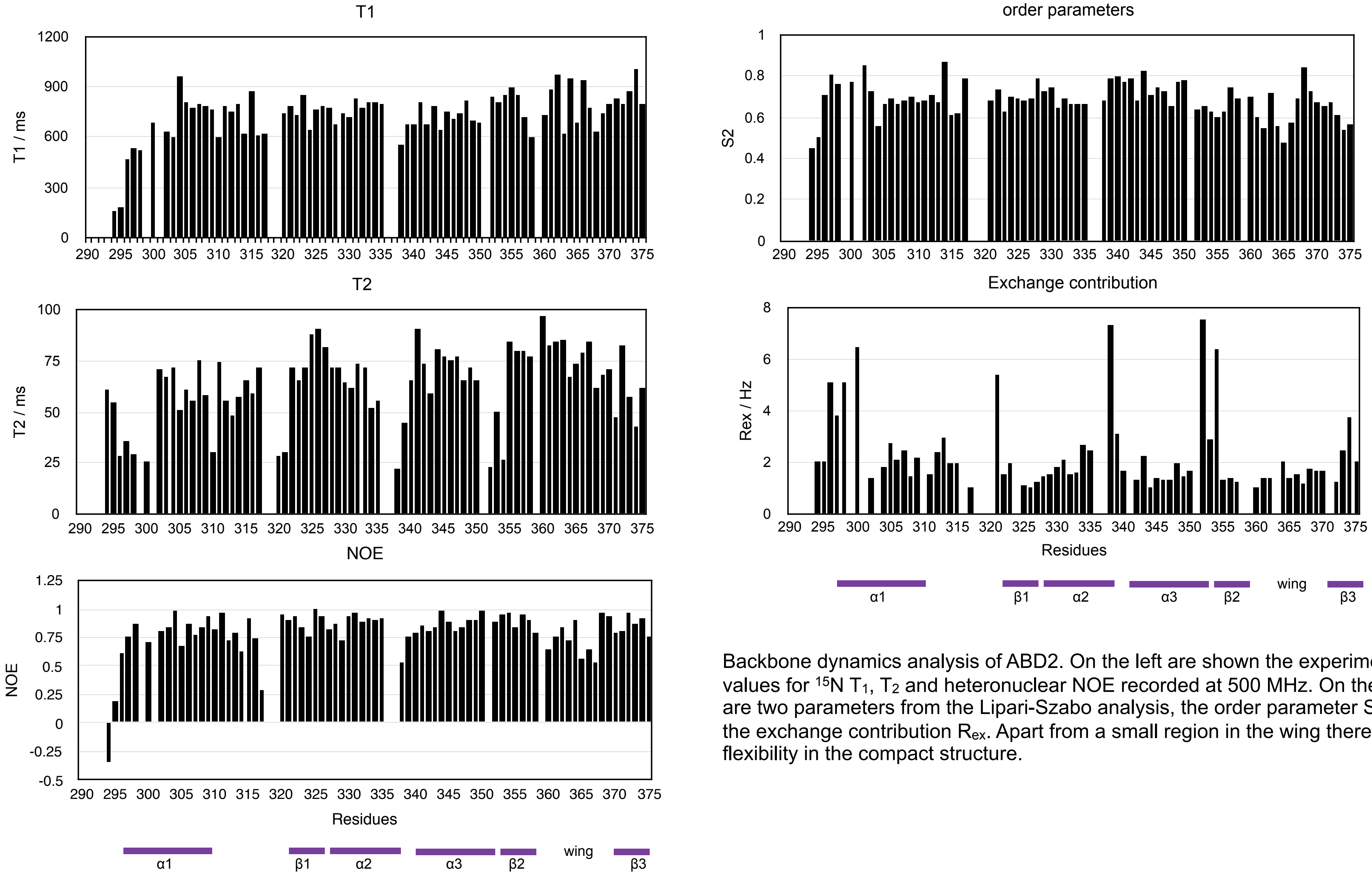

Backbone dynamics analysis of ABD2. On the left are shown the experimental values for ^{15}N T_1, T_2 and heteronuclear NOE recorded at 500 MHz. On the right are two parameters from the Lipari-Szabo analysis, the order parameter S^2 and the exchange contribution R_{ex}. Apart from a small region in the wing there is little flexibility in the compact structure.
