# Supplementary material for: The Cardiac Stress Response Factor Ms1 Can Bind to DNA and Has a Function in the Nucleus
Source: PLoS One. 2015 Dec 14;10(12):e0144614. doi: 10.1371/journal.pone.0144614 (PMC4682817; doi:10.1371/journal.pone.0144614)
Supplement: S3 Fig — Proteins that are not involved in DNA binding are marked with a blue star. Structures used in the superposition in Fig 1D are marked with red stars. (PDF) [file pone.0144614.s003.pdf]

S3 Fig

No:	Chain	Z	rmsd	lali	nres	%id	Description
1:	1sd4-B	5.3	2.8	59	122	8	MOLECULE: PENICILLINASE REPRESSOR; *
2:	2hr3-B	5.1	4.0	59	135	15	MOLECULE: PROBABLE TRANSCRIPTIONAL REGULATOR; *
3:	1sax-B	5.1	2.8	59	120	10	MOLECULE: METHICILLIN RESISTANCE REGULATORY PROTEIN MECI; *
4:	1p6r-A	5.1	2.4	58	82	21	MOLECULE: PENICILLINASE REPRESSOR;
5:	2d45-C	5.0	3.0	59	117	10	MOLECULE: METHICILLIN RESISTANCE REGULATORY PROTEIN MECI;
6:	1w1w-H	5.0	3.4	60	70	10	MOLECULE: STRUCTURAL MAINTENANCE OF CHROMOSOME 1;
7:	1w1w-F	5.0	3.6	62	70	10	MOLECULE: STRUCTURAL MAINTENANCE OF CHROMOSOME 1;
8:	1g3y-A	5.0	3.0	58	212	12	MOLECULE: DIPHTHERIA TOXIN REPRESSOR;
9:	1bi2-B	5.0	3.0	58	138	12	MOLECULE: DIPHTHERIA TOXIN REPRESSOR;
10:	3bpv-A	4.9	3.4	61	137	11	MOLECULE: TRANSCRIPTIONAL REGULATOR;
11:	2qww-A	4.9	3.5	63	146	14	MOLECULE: TRANSCRIPTIONAL REGULATOR, MARR FAMILY;
12:	2p7c-B	4.9	3.2	62	82	19	MOLECULE: STRAND 1 OF TWELVE BASE-PAIR DNA;
13:	2hr3-D	4.9	3.7	61	140	15	MOLECULE: PROBABLE TRANSCRIPTIONAL REGULATOR;
14:	2dtr	4.9	2.9	57	137	12	MOLECULE: DIPHTHERIA TOXIN REPRESSOR;
15:	2d45-B	4.9	2.9	59	119	10	MOLECULE: METHICILLIN RESISTANCE REGULATORY PROTEIN MECI;
16:	2acj-D	4.9	3.1	59	63	12	MOLECULE: DOUBLE-STRANDED RNA-SPECIFIC ADENOSINE DEAMINASE;
17:	1q1h-A	4.9	3.4	64	85	9	MOLECULE: TRANSCRIPTION FACTOR E;
18:	1okr-B	4.9	3.1	59	122	10	MOLECULE: METHICILLIN RESISTANCE REGULATORY PROTEIN MECI;
19:	1okr-A	4.9	3.1	59	120	10	MOLECULE: METHICILLIN RESISTANCE REGULATORY PROTEIN MECI;
20:	1g3t-B	4.9	3.0	58	138	12	MOLECULE: DIPHTHERIA TOXIN REPRESSOR;
21:	1fwz-A	4.9	2.8	57	211	12	MOLECULE: DIPHTHERIA TOXIN REPRESSOR;
22:	1bi3-B	4.9	2.9	57	137	12	MOLECULE: DIPHTHERIA TOXIN REPRESSOR;
23:	2pfb-B	4.8	3.2	57	123	21	MOLECULE: TRANSCRIPTIONAL REGULATOR OHRR;
24:	2gxb-B	4.8	3.0	58	63	12	MOLECULE: DOUBLE-STRANDED RNA-SPECIFIC ADENOSINE DEAMINASE;
25:	1g3t-A	4.8	3.0	57	215	12	MOLECULE: DIPHTHERIA TOXIN REPRESSOR;
26:	1bi3-A	4.8	2.8	56	212	13	MOLECULE: DIPHTHERIA TOXIN REPRESSOR;
27:	3cta-A	4.7	2.7	59	187	14	MOLECULE: RIBOFLAVIN KINASE; *
28:	3c18-C	4.7	2.6	58	290	16	MOLECULE: NUCLEOTIDYLTRANSFERASE-LIKE PROTEIN; *
29:	3c18-A	4.7	2.6	58	290	16	MOLECULE: NUCLEOTIDYLTRANSFERASE-LIKE PROTEIN;
30:	2qqa-A	4.7	2.9	57	218	12	MOLECULE: DIPHTHERIA TOXIN REPRESSOR;
31:	2pex-B	4.7	3.1	55	137	22	MOLECULE: TRANSCRIPTIONAL REGULATOR OHRR;
32:	2gxb-A	4.7	3.1	58	62	12	MOLECULE: DOUBLE-STRANDED RNA-SPECIFIC ADENOSINE DEAMINASE;
33:	2fe3-B	4.7	3.1	64	143	13	MOLECULE: PEROXIDE OPERON REGULATOR;
34:	2fe3-A	4.7	2.8	62	141	13	MOLECULE: PEROXIDE OPERON REGULATOR;
35:	1xsd-A	4.7	2.9	59	125	8	MOLECULE: PENICILLINASE REPRESSOR;
36:	2qww-H	4.6	3.4	59	143	15	MOLECULE: TRANSCRIPTIONAL REGULATOR, MARR FAMILY;
37:	2qww-G	4.6	3.3	59	140	15	MOLECULE: TRANSCRIPTIONAL REGULATOR, MARR FAMILY;
38:	2qww-B	4.6	3.6	61	144	15	MOLECULE: TRANSCRIPTIONAL REGULATOR, MARR FAMILY;
39:	2pfb-A	4.6	3.0	57	129	23	MOLECULE: TRANSCRIPTIONAL REGULATOR OHRR;
40:	2jt1-A	4.6	3.3	60	71	13	MOLECULE: PEFI PROTEIN;
41:	2it0-D	4.6	2.9	56	137	16	MOLECULE: IRON-DEPENDENT REPRESSOR IDER;
42:	2it0-C	4.6	3.1	58	137	17	MOLECULE: IRON-DEPENDENT REPRESSOR IDER;
43:	2hzt-C	4.6	4.2	61	95	11	MOLECULE: PUTATIVE HTH-TYPE TRANSCRIPTIONAL REGULATOR YTCD;
44:	2hr3-A	4.6	4.2	62	139	16	MOLECULE: PROBABLE TRANSCRIPTIONAL REGULATOR;
45:	2frh-A	4.6	2.9	59	127	8	MOLECULE: STAPHYLOCOCCAL ACCESSORY REGULATOR A;
46:	2d45-D	4.6	2.9	59	115	10	MOLECULE: METHICILLIN RESISTANCE REGULATORY PROTEIN MECI;
47:	2acj-C	4.6	3.0	58	66	12	MOLECULE: DOUBLE-STRANDED RNA-SPECIFIC ADENOSINE DEAMINASE;
48:	1u8r-I	4.6	3.0	57	221	16	MOLECULE: IRON-DEPENDENT REPRESSOR IDER;
49:	1u8r-H	4.6	3.2	58	222	17	MOLECULE: IRON-DEPENDENT REPRESSOR IDER;
50:	1u8r-C	4.6	3.3	58	221	17	MOLECULE: IRON-DEPENDENT REPRESSOR IDER;

Top 50 hits of DALI search of the PDB with the structure of ABD2. Proteins that are not involved in DNA binding are marked with a blue star. Structures used in the superposition in Fig. 1D are marked with red stars.
