# Supplementary material for: The Cardiac Stress Response Factor Ms1 Can Bind to DNA and Has a Function in the Nucleus
Source: PLoS One. 2015 Dec 14;10(12):e0144614. doi: 10.1371/journal.pone.0144614 (PMC4682817; doi:10.1371/journal.pone.0144614)
Supplement: S4 Fig — The antibody (aABD2chn) was generated for detection of endogenous Ms1. It was validated by western blots with myc-tagged full length Ms1 expressed in COS cells (left); western blots of endogenous Ms1 in muscle tissue extracts with untreated and blocked antibody (lower left); western blots of overexpressed myc-tagged full length Ms1 in HeLa cells (below); (PDF) [file pone.0144614.s004.pdf]

S4 Fig

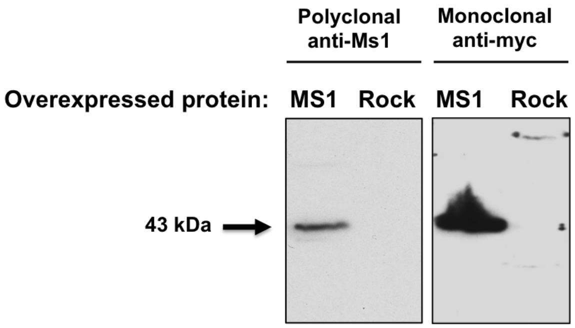

Overexpression of myc-tagged Ms1 in COS cells using myc-tagged Rock as a control

Validation of new antibody against mouse ABD2. The antibody (aABD2chn) was generated for detection of endogenous Ms1. It was validated by western blots with myc-tagged full length Ms1 expressed in COS cells (left); western blots of endogenous Ms1 in muscle tissue extracts with untreated and blocked antibody (lower left); western blots of overexpressed myc-tagged full length Ms1 in HeLa cells (below); immunofluorescence detection of transfected full length myc-tagged Ms1 in HeLa cells using anti-myc as well as aABD2chn antibodies (S4B, left) and immunofluorescence detection of endogenous Ms1 in NRCs with untreated and blocked aABD2chn (S4B right).

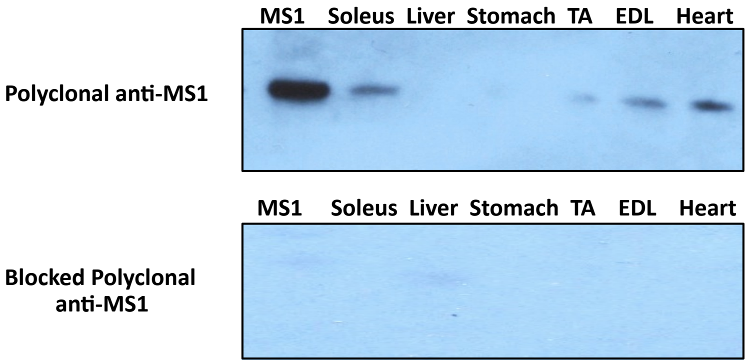

Detection of endogenous Ms1 in s selection of rat tissues using antibody directly and after blocking with original antigen. TA=tibialis anterior, EDL=extensor digitorum longus.

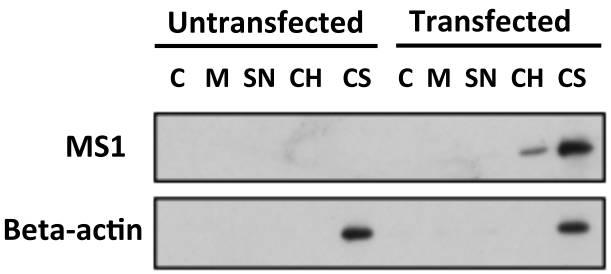

Western blot detection of overexpressed myc-tagged Ms1 in HeLa cells in subcellular fractions. C=cytoplasmic, M=membrane, SN=soluble nuclear, CH=chromatin, CS=cytoskeleton
