# Supplementary figures and images for: The Cardiac Stress Response Factor Ms1 Can Bind to DNA and Has a Function in the Nucleus
Source: PLoS One. 2015 Dec 14;10(12):e0144614. doi: 10.1371/journal.pone.0144614 (PMC4682817; doi:10.1371/journal.pone.0144614)

HeLa cells

NRCs

S5 Fig

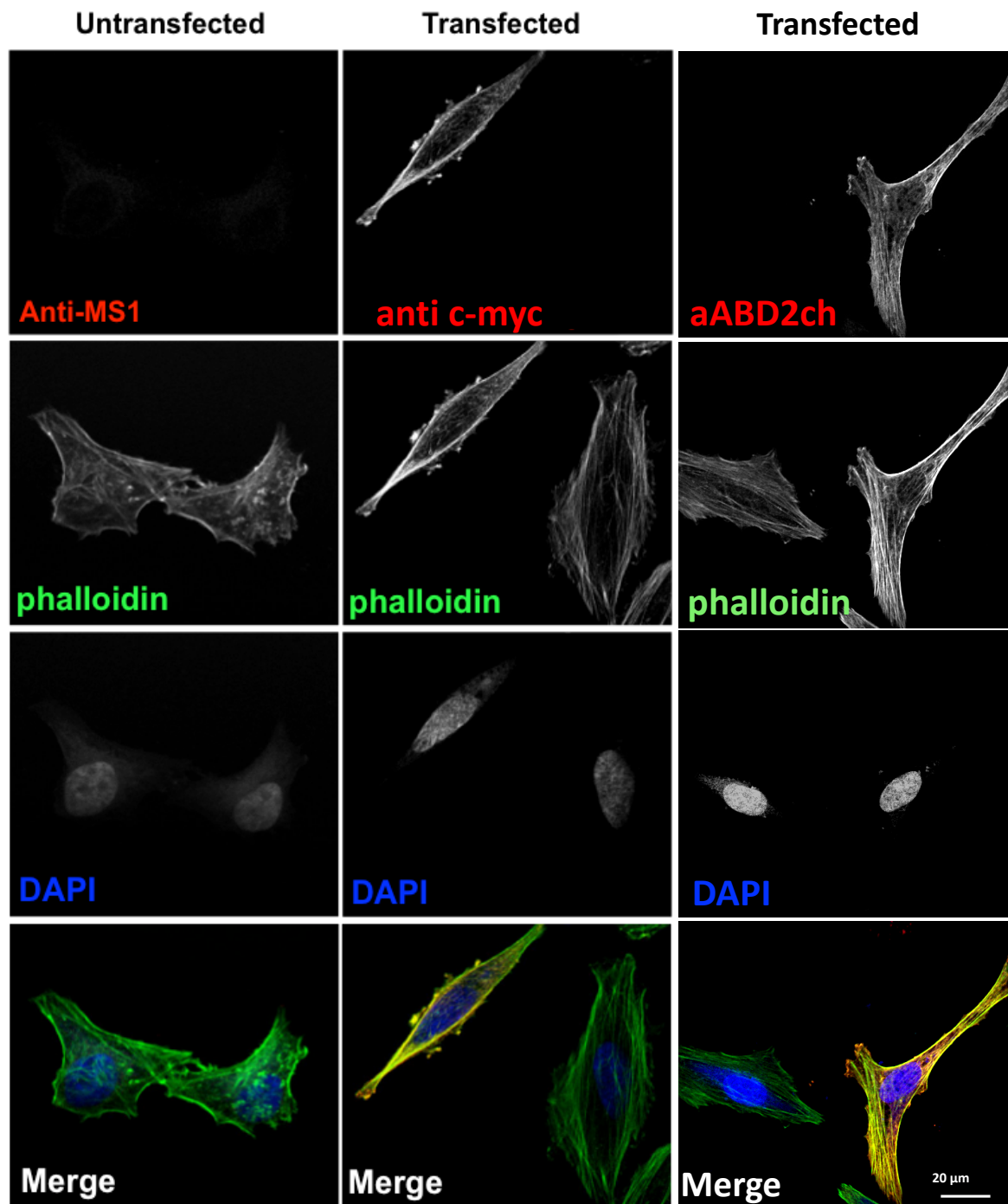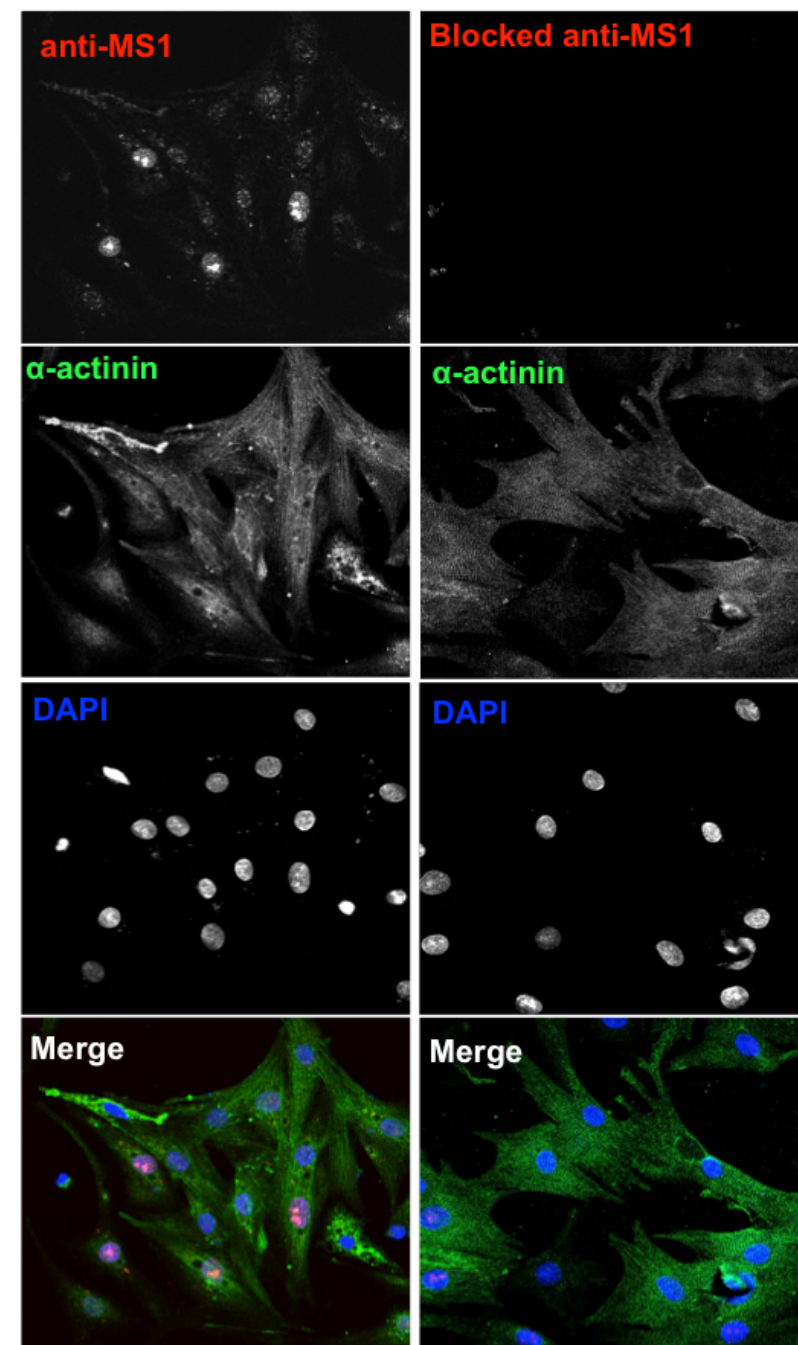

Supplement: S5 Fig — Immunofluorescence detection of transfected full length myc-tagged Ms1 in HeLa cells using anti-myc as well as aABD2chn antibodies (left) and immunofluorescence detection of endogenous Ms1 in NRCs with untreated and blocked aABD2chn (right). (PDF) [file pone.0144614.s005.pdf]
