# Supplementary material for: The Cardiac Stress Response Factor Ms1 Can Bind to DNA and Has a Function in the Nucleus
Source: PLoS One. 2015 Dec 14;10(12):e0144614. doi: 10.1371/journal.pone.0144614 (PMC4682817; doi:10.1371/journal.pone.0144614)
Supplement: S6 Fig — A significant increase in stability of > 7°C is seen for the addition of 23 amino acids at the N-terminus of ABD2.This addition leads to considerable chemical shift changes around helix one to which it would need to pack to allow the AT-hook to reach the DNA. (PDF) [file pone.0144614.s006.pdf]

S6 Fig

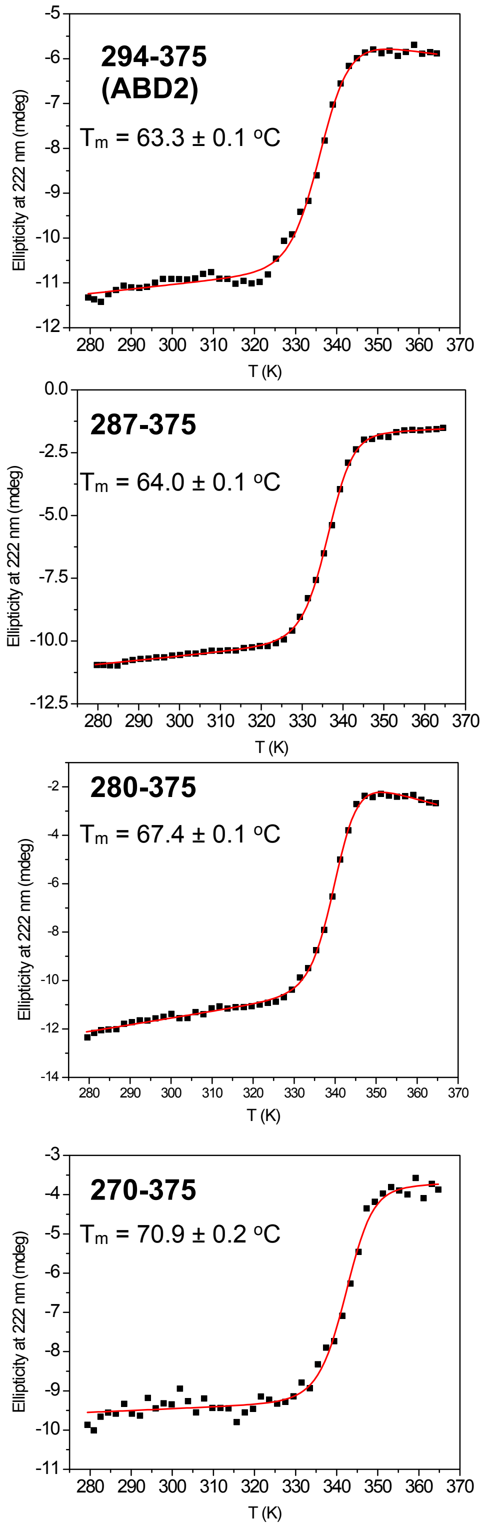

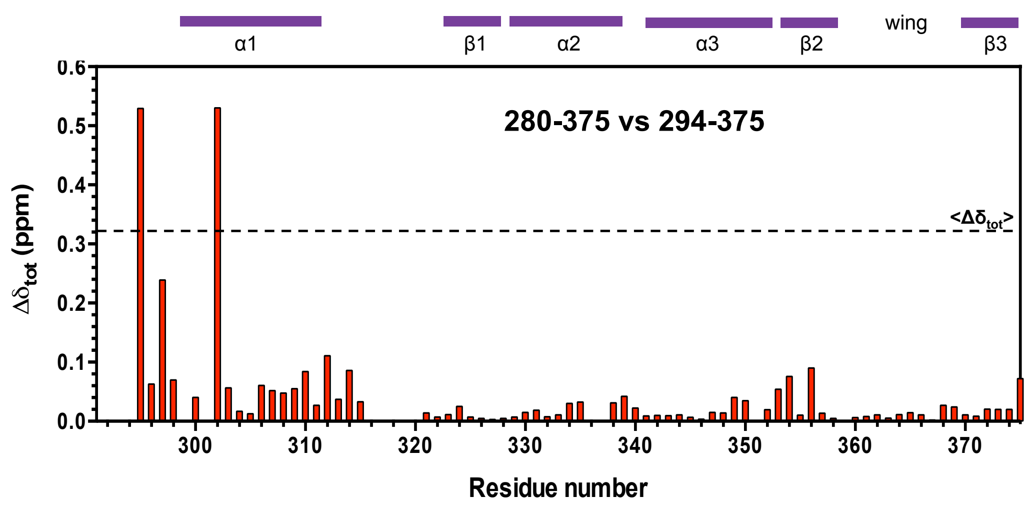

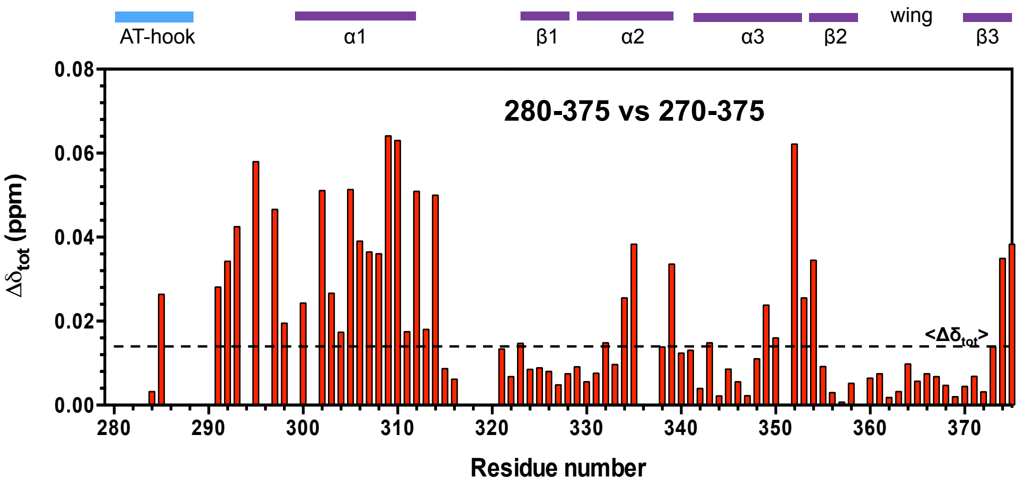

Effects of potential interactions of the N-terminal extension of ABD2 on stability and NMR spectra of ABD2. A significant increase in stability of > 7 °C is seen for the addition of 23 amino acids at the N-terminus of ABD2. This addition leads to considerable chemical shift changes around helix one to which it would need to pack to allow the AT-hook to reach the DNA.
